# Supplementary material for: Electrifying long-haul freight trucks reduces societal costs in the United States
Source: Nat Commun. 2025 Dec 12;17:468. doi: 10.1038/s41467-025-67161-1 (PMC12800144; doi:10.1038/s41467-025-67161-1)
Supplement: Supplementary file 1 — Supplementary Information File [file 41467_2025_67161_MOESM1_ESM.pdf]

Supplemental Information

Electrifying Long-Haul Freight Trucks Reduces Societal Costs in the United States

Jason Porzio[1,2], Wilson McNeil[1,2], Fan Tong[3,4,5], Scott Moura[2], Maximilian Auffhammer[6,7] **, Corinne D. Scown[1*]

[1] Energy and Biosciences Institute, University of California, Berkeley; Berkeley, California 94720, United States
[2] Civil and Environmental Engineering Department, University of California, Berkeley; Berkeley, California 94720, United States
[3] School of Economics and Management, Beihang University; Beijing 100191, People's Republic of China
[4] Lab for Low-carbon Intelligent Governance, Beihang University; Beijing 100191, People's Republic of China
[5] Peking University Ordos Research Institute of Energy; Ordos City, Inner Mongolia 017000, People's Republic of China
[6] Department of Agricultural and Resource Economics, University of California, Berkeley; Berkeley, California 94720, United States
[7] National Bureau of Economic Research; Cambridge, Massachusetts 02138, United States
**Correspondence: auffhammer@berkeley.edu
*Correspondence: cscown@berkeley.edu

The PDF file includes:

Discussion S1 to S2
Figs. S1 to S49
Tables S1 to S13
References

Discussion S1

This study employs a marginal short-run generation model when estimating generator response, following the precedent set throughout literature when evaluating electricity impacts associated with small quantities of electric vehicles inducing relatively small changes in generator behavior. [1–10] In this study, the induced generation demand from BE-HDVs relative to the total quantity of generation nationwide is near negligible and will rely on marginal generation resources (i.e. not baseload), allowing for the use of a marginal short-run generation model.

For studies that examine dramatic changes to electricity demand or the impacts associated with average electricity demand, a short-run marginal generational model may mischaracterize generation impacts. Gagnon and Cole[9] employ 3 models alongside observed data to find that short-run marginal emission factors (EFs) are roughly 2-3 times higher than average EFs and long-run marginal EFs. This can largely be attributed to the increasing tendency to use coal generation and reliance on less efficient generators to meet marginal electricity demand, resulting in marginal EFs that have been slightly increasing over time despite average emissions decreasing.[11,12] Importantly, Holland et al. finds that EFs of marginal generators are substantially higher than EFs of average generators of the same fuel type due to differences in efficiency attributable to lower utilization rates.

Future studies regarding the electrification should carefully consider whether a marginal short-run generation model accurately characterizes their load type and corresponding generator response, as well as whether emissions associated with this modeling choice will substantially vary over time.

Discussion S2

Our Markov Chain Monte Carlo performs 100 runs (i.e. simulates a fleet of 100 trucks) in order to estimate the average marginal impact of HDV electrification with Li-ion batteries per VMT. This fleet size is selected purely due to computational limitations and the time it takes to run our model iteratively. Additionally, the short-run marginal grid model we employ defines marginal generators for each region and each hour of the year across different scenarios, but it does not specify exactly how much additional capacity is available at each generator before a different type of generator would be called upon. This is a fundamental limitation of the Cambium data we rely on from the NREL scenarios. However, given the relatively small instantaneous demand from a single truck charging (on the order of magnitude of tens to hundreds of kWs), and the wide dispersal of vehicles across the US, our results should be representative of long-haul HDV electrification at much larger scales than 100 trucks (likely for fleets several orders of magnitude larger). Nevertheless, we cannot say with certainty the maximum scale beyond which our inherently linear per-VMT results would no longer be representative of realistic grid responses and emissions.

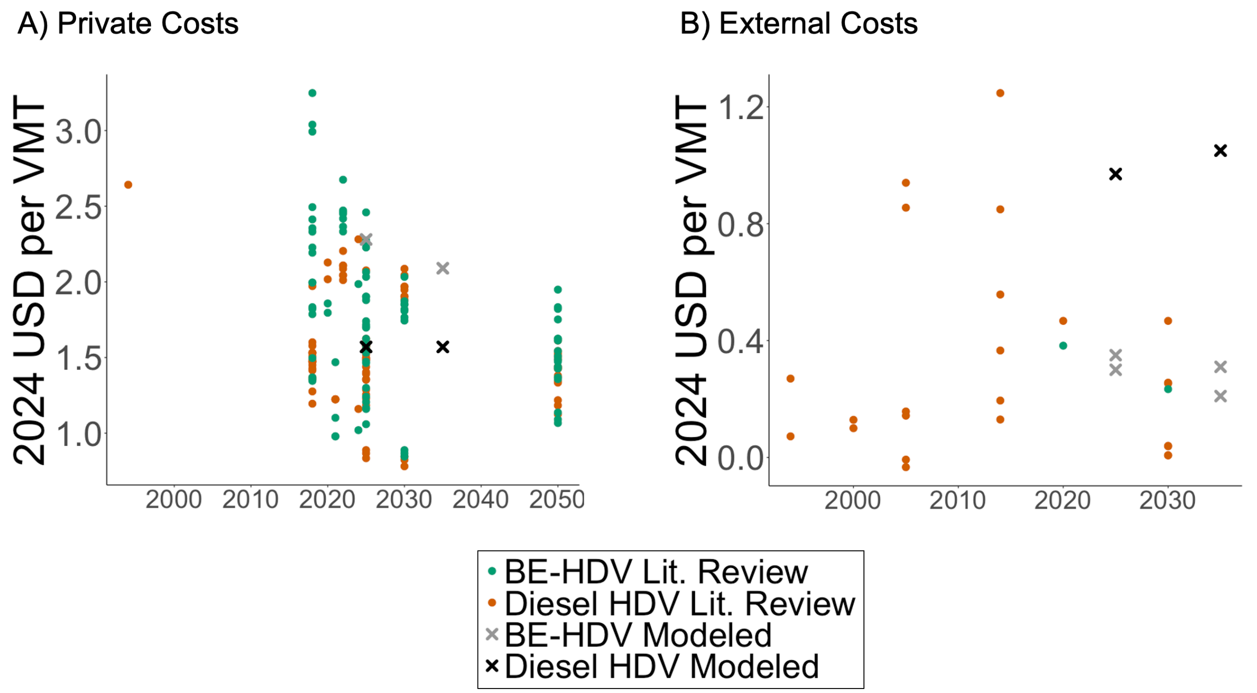

Fig. S1. Literature review and comparison of private costs and external costs of BE-HDVs and diesel HDVs.[13–24]

Table S1. Monetized human health damages of BE-HDV in cents per kWh.

Year	Renewable Energy Cost Scenario	Human Health Damages (Cents per kWh)	Human health Damages Standard Deviation (Cents per kWh)
2025	Low RE	5.79	0.72375
2025	High RE	6.57	0.82125
2035	Low RE	2.34	0.29250
2035	High RE	4.65	0.58125

Table S2. Monetized human health damages of electricity in Cohon et al.[22]

Fuel	Methodology	2024 US Cents per kWh		
		5th Percentile	Mean	95th Percentile
Coal	Unweighted	0.81	6.69	20.64
Coal	Weighted by net generation	0.29	4.86	18.24
Natural Gas	Unweighted	0.0067	0.65	2.58
Natural Gas	Weighted by net generation	0.0015	0.24	0.836

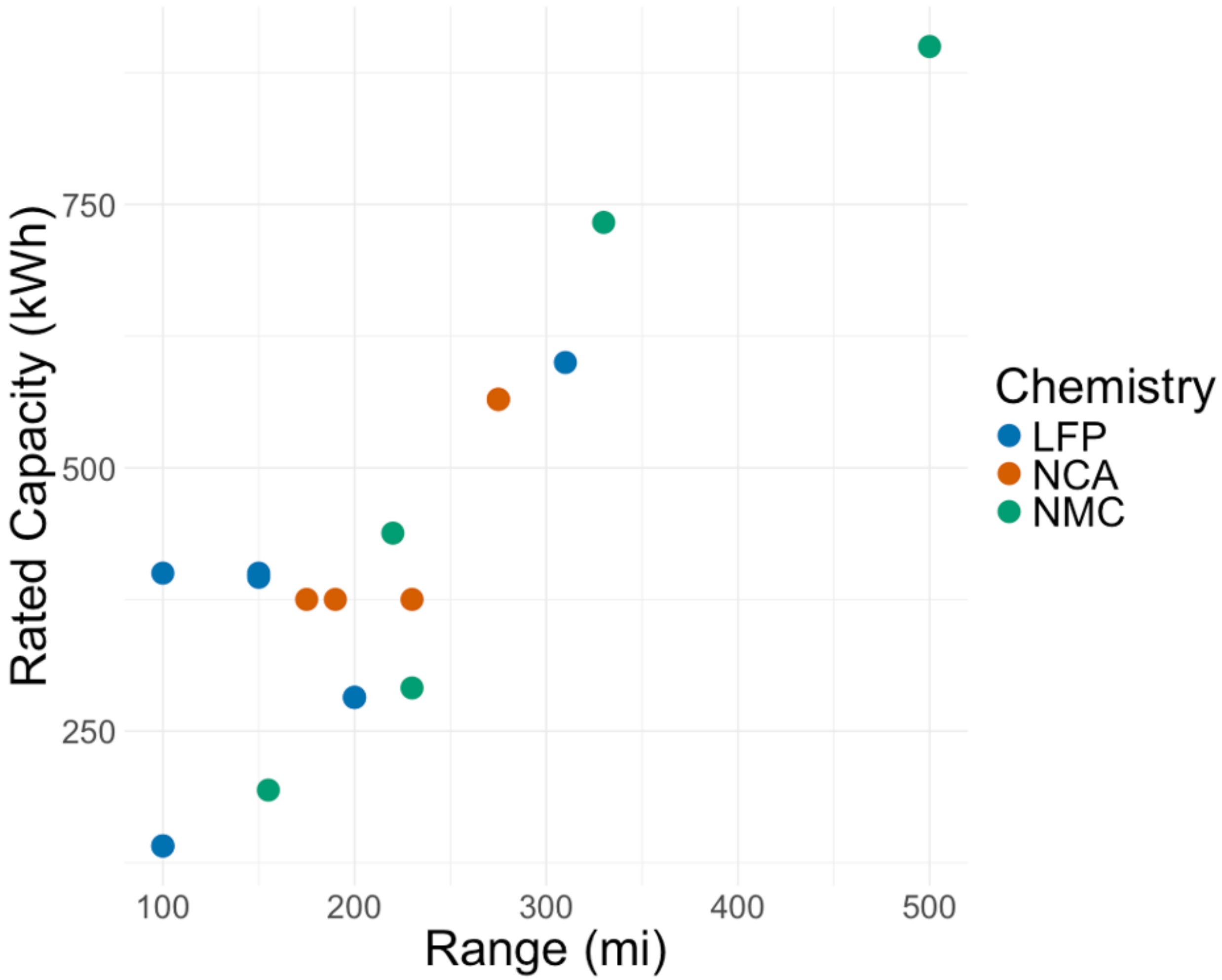

Fig S2. 2024 market battery electric class 8 trucks - rated capacity vs range. All data collected via independent review of available class 8 trucks to date.

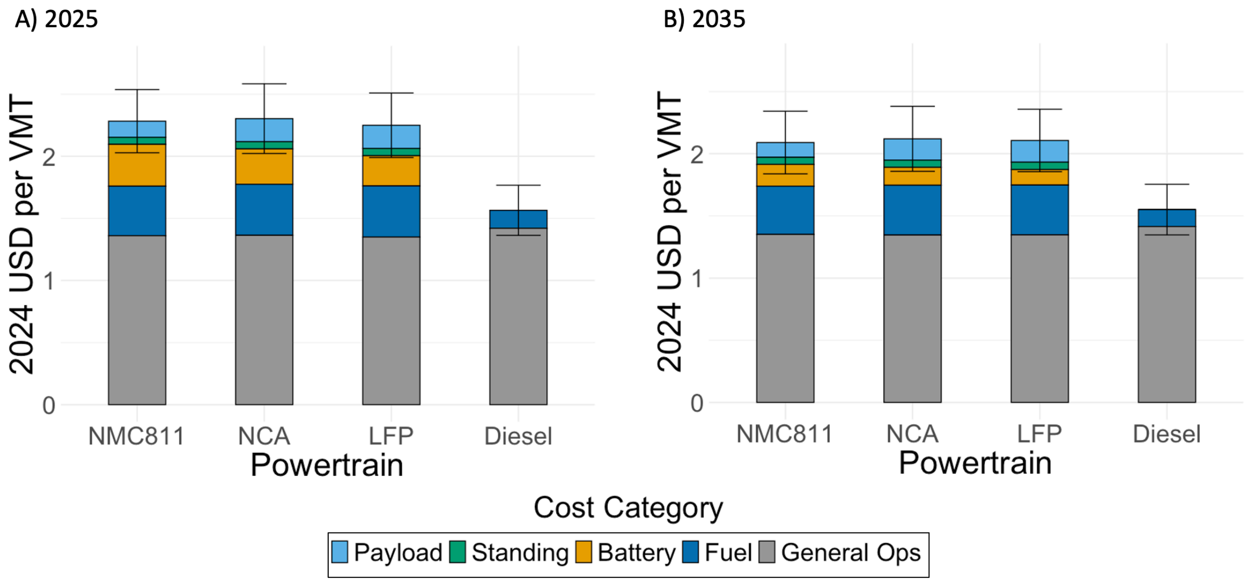

Fig. S3 Net present value of private costs of BE-HDVs and diesel HDVs performing long-haul freight in varying years and renewable energy cost scenarios with a 2% discount rate. (**A**) Visualized private costs in 2025. (**B**) Visualized private costs in 2035.

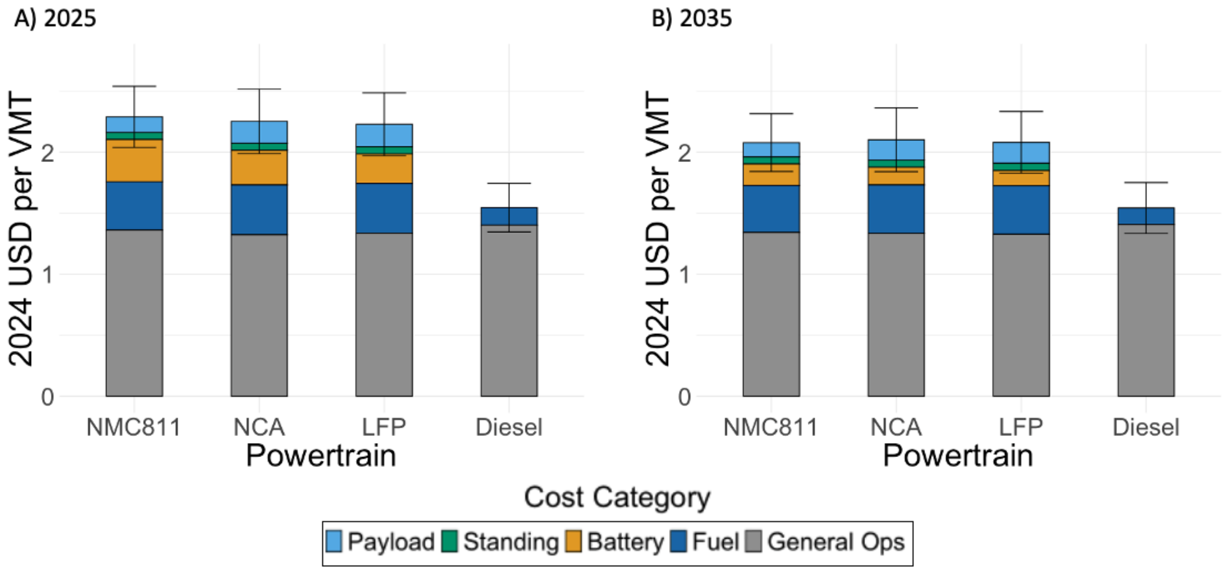

Fig S4. Private costs of BE-HDVs and diesel HDVs performing long-haul freight in varying years and renewable energy cost scenarios with a 2.5% discount rate. a) Visualized private costs in 2025. b) Visualized private costs in 2025.

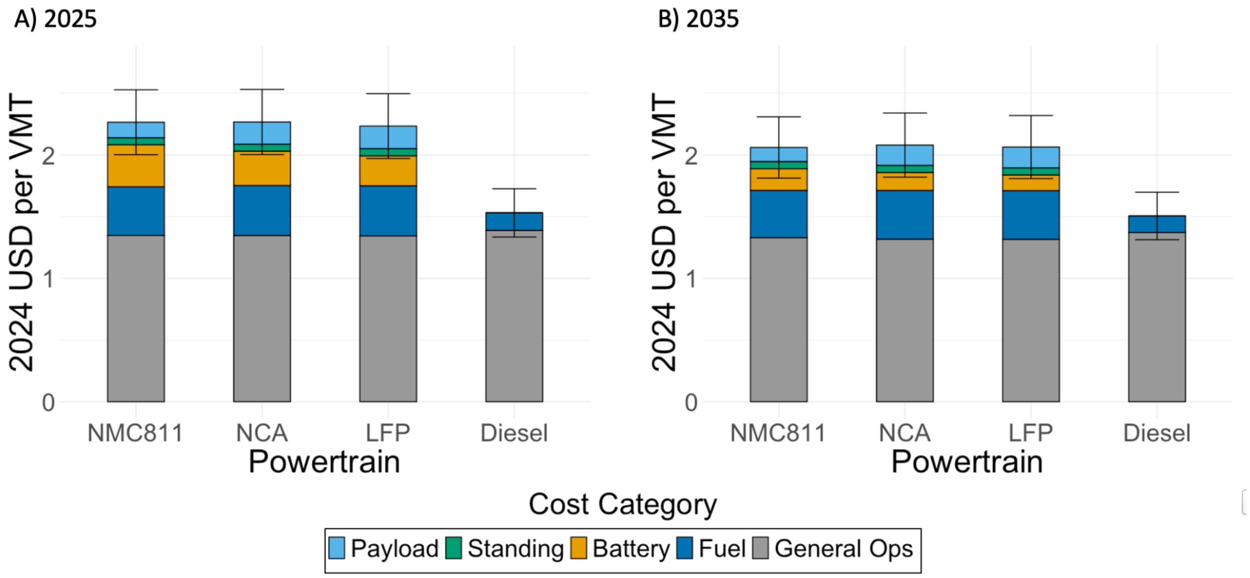

Fig S5. Private costs of BE-HDVs and diesel HDVs performing long-haul freight in varying years and renewable energy cost scenarios with a 3% discount rate. a) Visualized private costs in 2025. b) Visualized private costs in 2035.

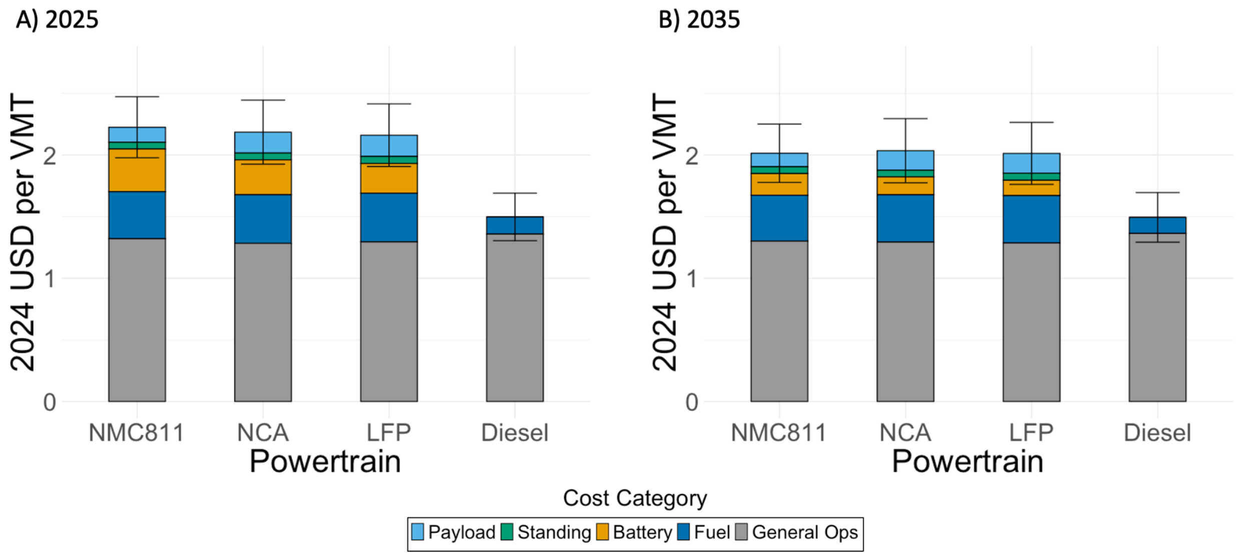

Fig. S6 Net present value of private costs of BE-HDVs and diesel HDVs performing long-haul freight in varying years and renewable energy cost scenarios with a 5% discount rate. (**A**) Visualized private costs in 2025. (**B**) Visualized private costs in 2035.

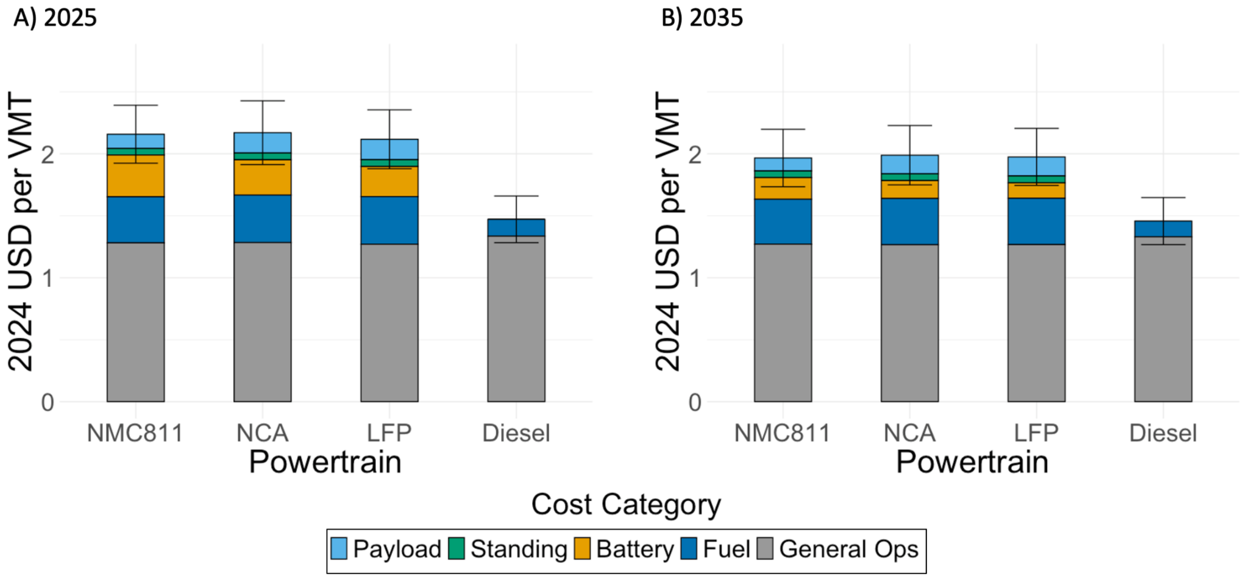

Fig S7. Private costs of BE-HDVs and diesel HDVs performing long-haul freight in varying years and renewable energy cost scenarios with a 7% discount rate. a) Visualized private costs in 2025. b) Visualized private costs in 2025.

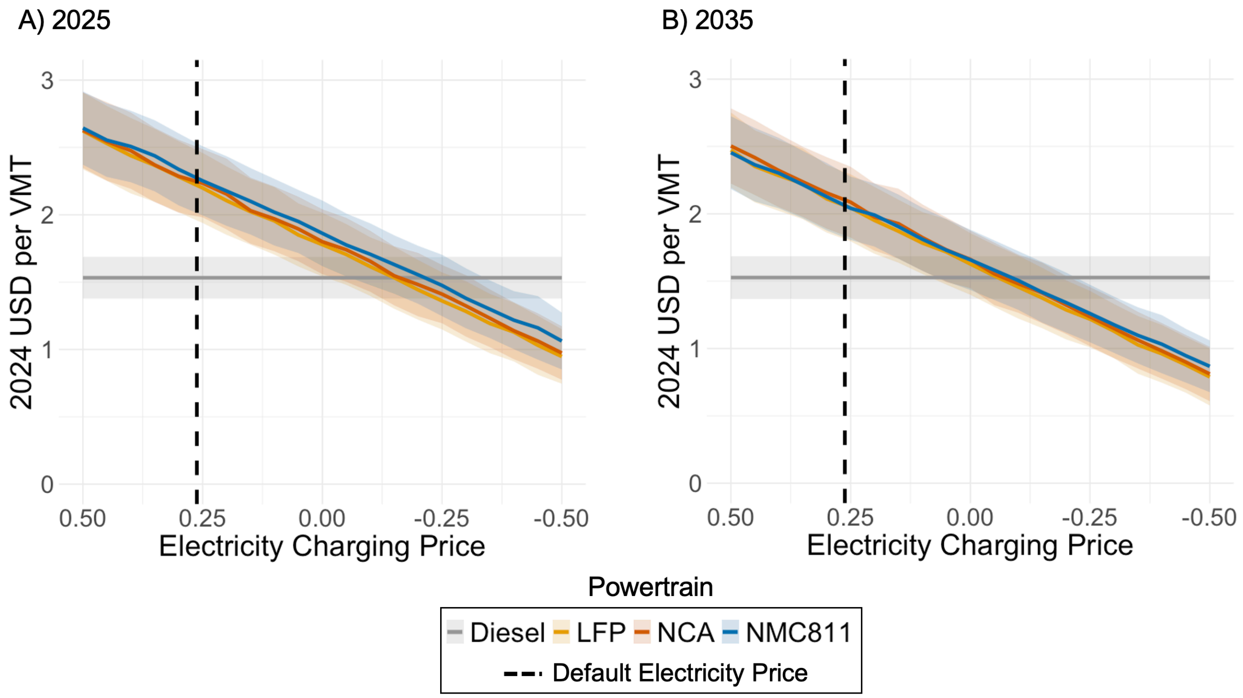

Fig S8. Sensitivity analysis on the impact of electricity price on private costs of BE-HDVs and diesel HDVs performing long-haul freight in varying years and renewable energy cost scenarios with a 3% discount rate. A) Visualized private costs in 2025. B) Visualized private costs in 2035.

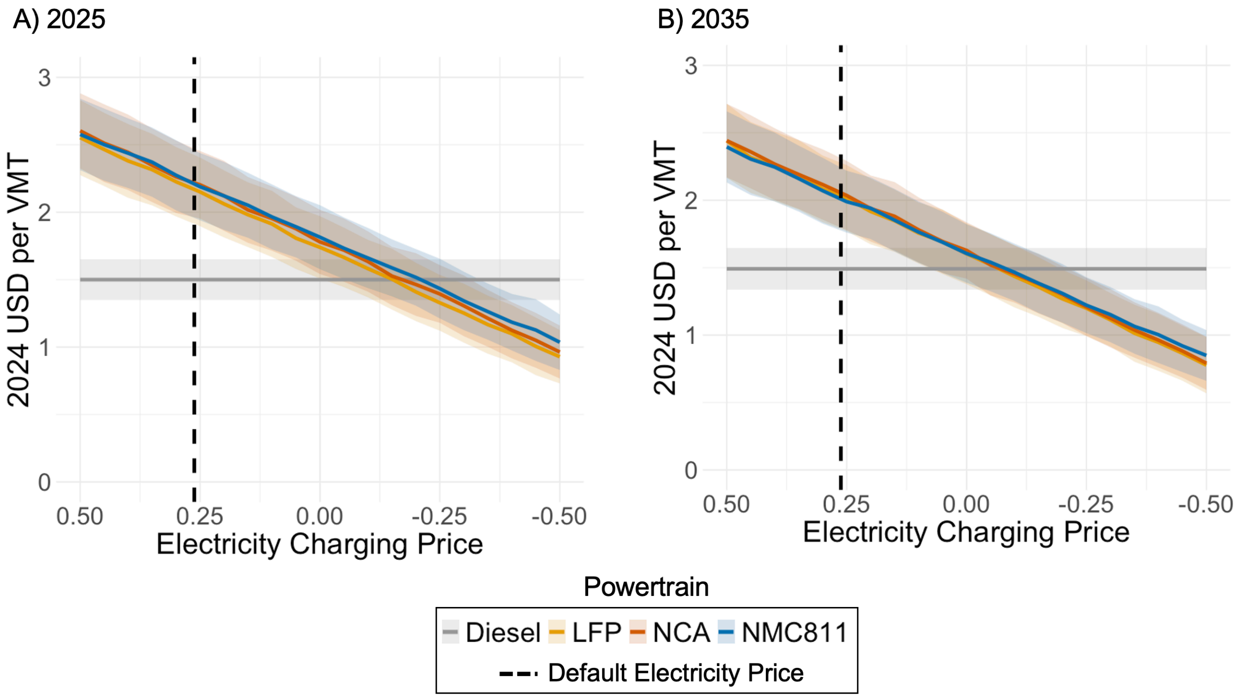

Fig. S9 Sensitivity analysis on the impact of electricity price on private costs of BE-HDVs and diesel HDVs performing long-haul freight in varying years and renewable energy cost scenarios with a 5% discount rate. A) Visualized private costs in 2025. B) Visualized private costs in 2035.

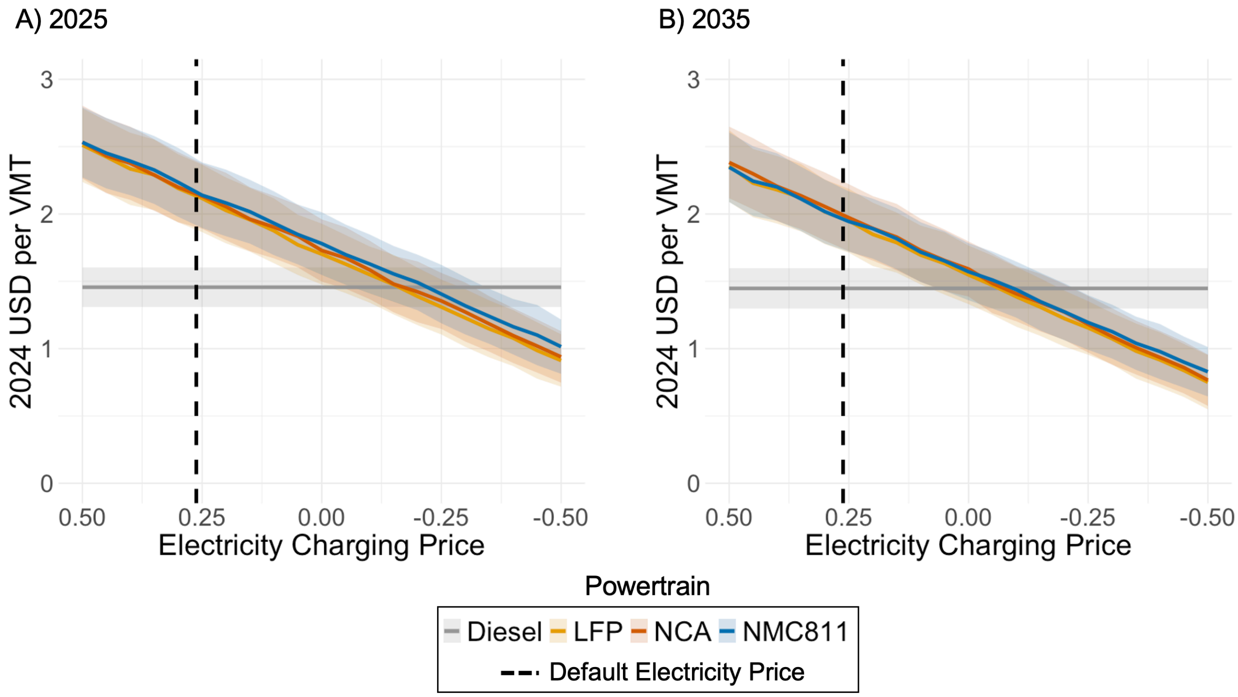

Fig S10. Sensitivity analysis on the impact of electricity price on private costs of BE-HDVs and diesel HDVs performing long-haul freight in varying years and renewable energy cost scenarios with a 7% discount rate. A) Visualized private costs in 2025. B) Visualized private costs in 2035.

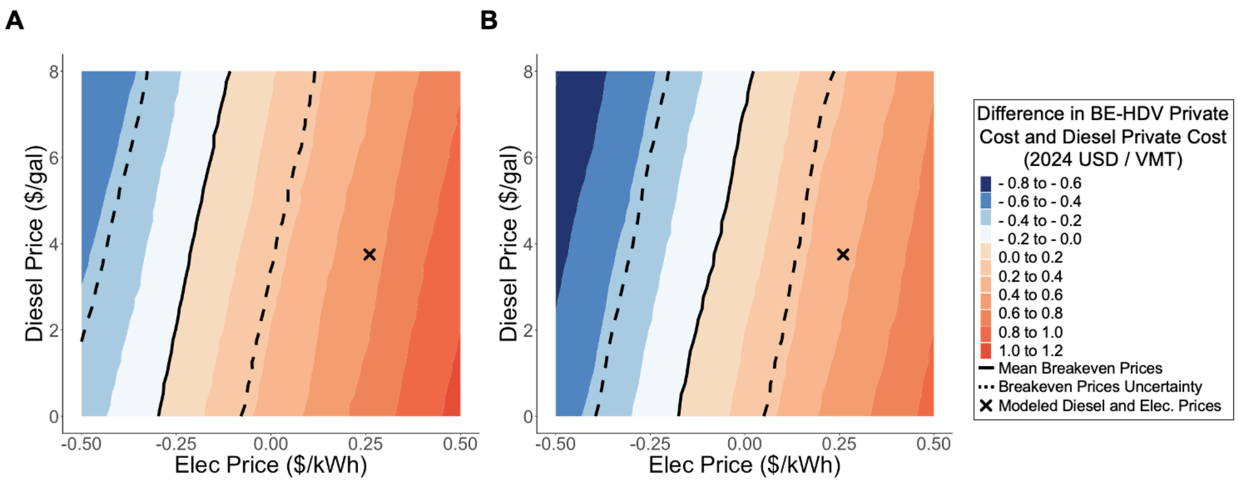

Fig. S11. Sensitivity of the difference in BE-HDV private costs and diesel private costs to electricity and diesel prices with a 3% for (A) 2025 and (B) 2035. The high and low breakeven prices represent the range across two standard deviations for private costs of BE-HDVs and diesel HDVs.

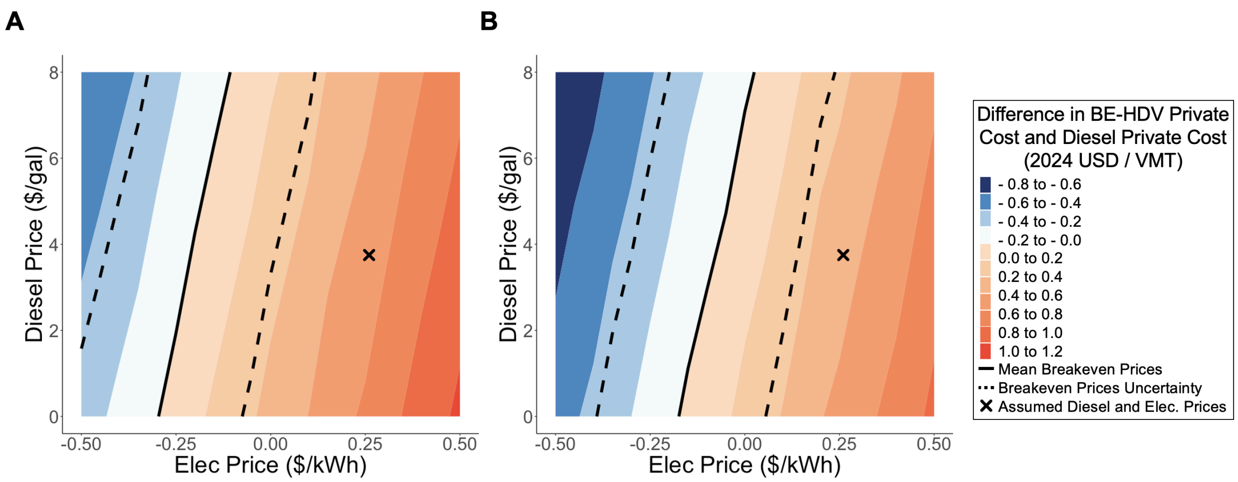

Fig. S12. Sensitivity of the difference in BE-HDV private costs and diesel private costs to electricity and diesel prices with a 5% for (A) 2025 and (B) 2035. The high and low breakeven prices represent the range across two standard deviations for private costs of BE-HDVs and diesel HDVs.

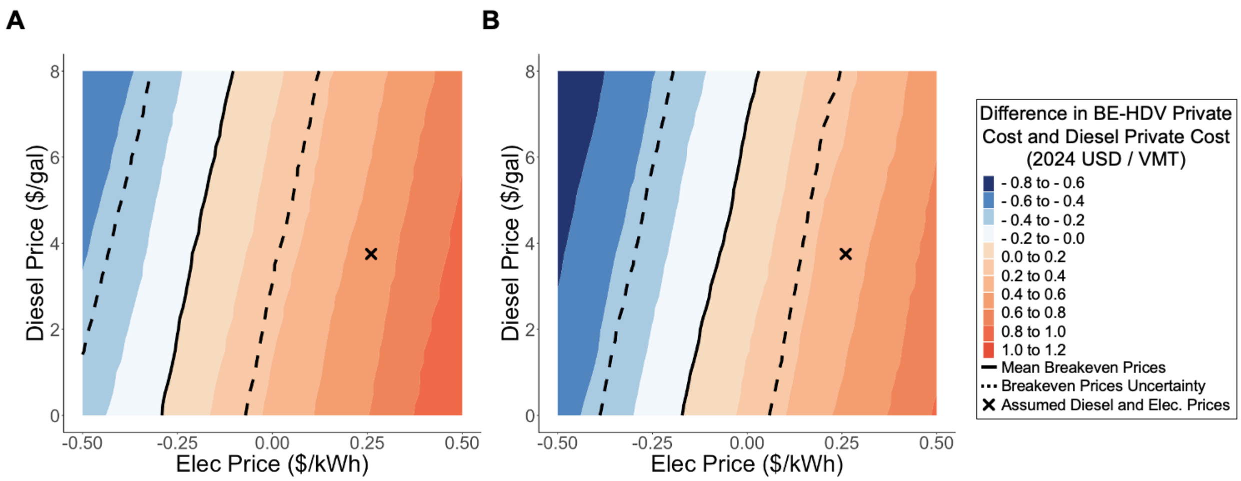

Fig. S13. Sensitivity of the difference in BE-HDV private costs and diesel private costs to electricity and diesel prices with a 7% for (A) 2025 and (B) 2035. The high and low breakeven prices represent the range across two standard deviations for private costs of BE-HDVs and diesel HDVs.

Table S3. Truck design parameters by performance scenario.

Parameter	Li-ion Class 8 Truck	Diesel Class 8 Truck
R_{Br} [%]	7.5	-
h_{BW} [%]	0.85	-
h_{Br} [%]	0.97	0.97
h_{GB} [%]	0.95	-
h_{E} [%]	-	0.42
h_{TW} [%]	-	0.9
C_{D}	0.63	0.63
A [m^2]	5.4	5.4
C_{rr}	0.0055725	0.0055725
m_{B} [kg]	Var. by Chem.	0
m_{V} [kg]	8,767	13,267
GVWR [lbs]	82,000	80,000
P_{AC} [kW]	If ref.* 8 kW<br>Else 2 kW	If ref.* 8 kW<br>Else 2 kW
Full Rated Capacity [kWh]	1000	-
Available Capacity [kWh]	85	-
Range [mi]	560	-

* Refrigerated trailer

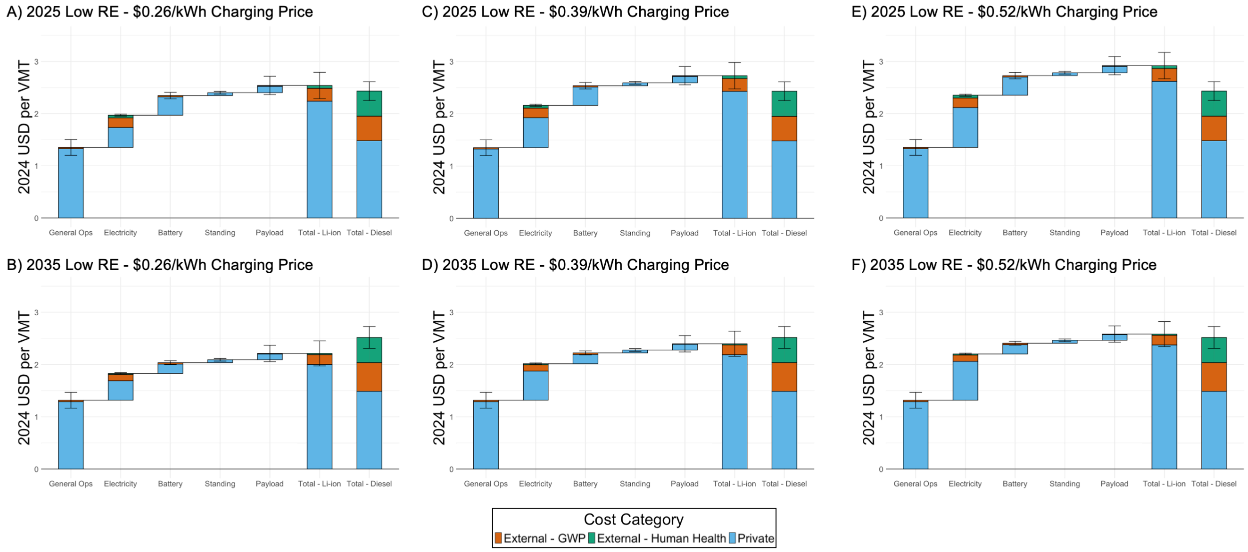

Fig. S14. Social costs under a low renewable cost scenario for a variety of years and electricity prices with a 2% discount rate.

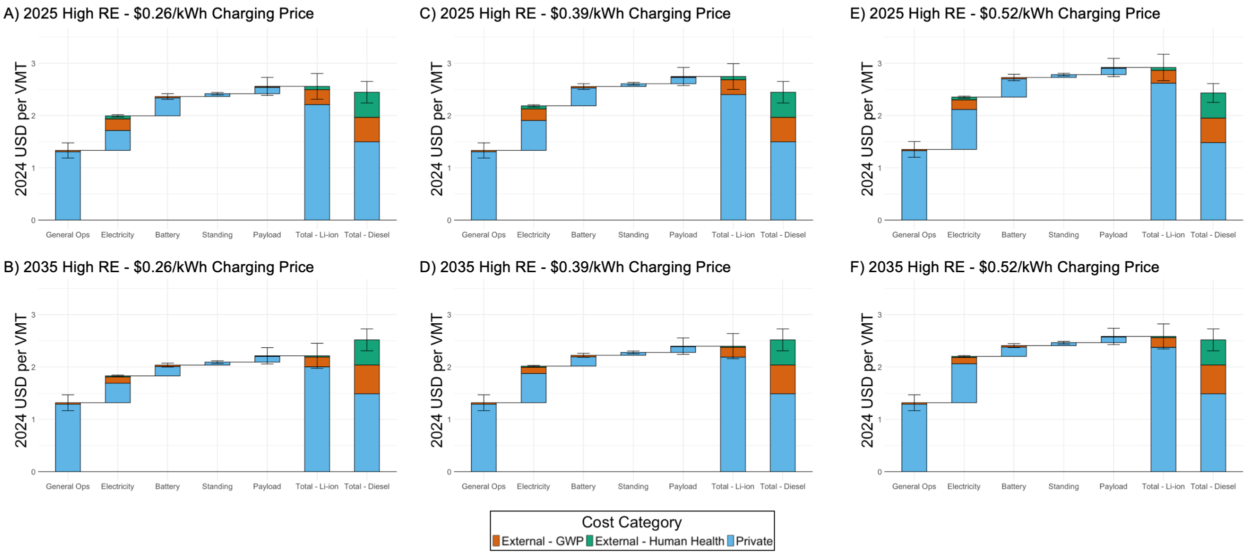

Fig. S15. Social costs under a high renewable cost scenario for a variety of years and electricity prices with a 2% discount rate.

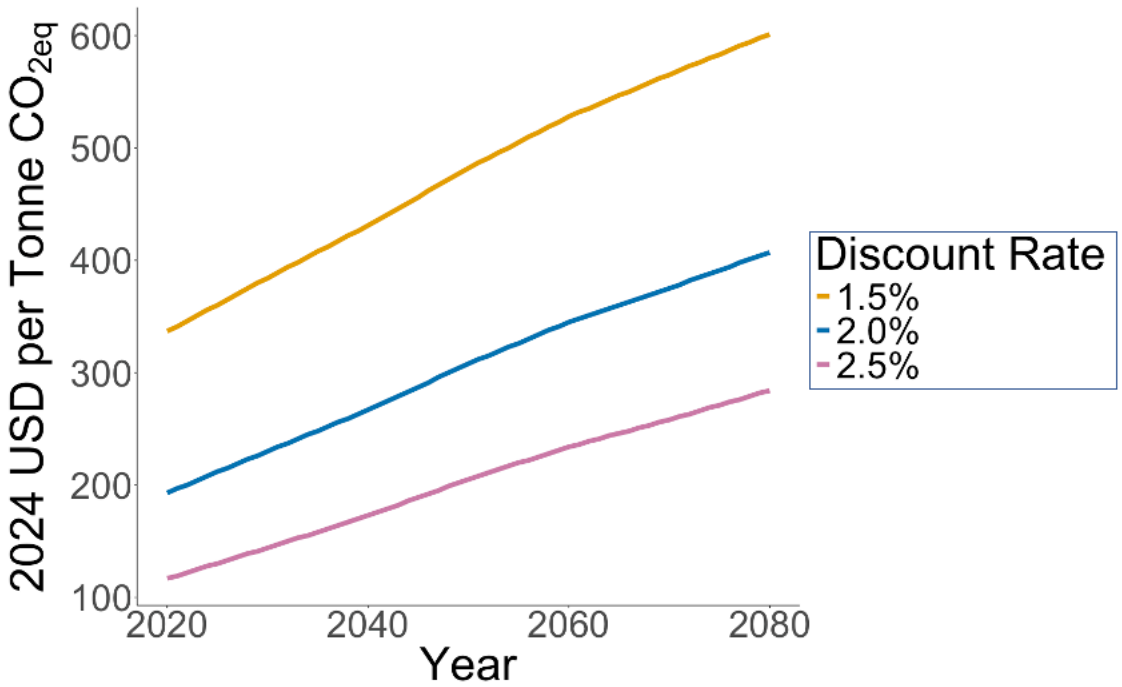

Fig. S16. Social cost of carbon overtime with different discount rates.[25]

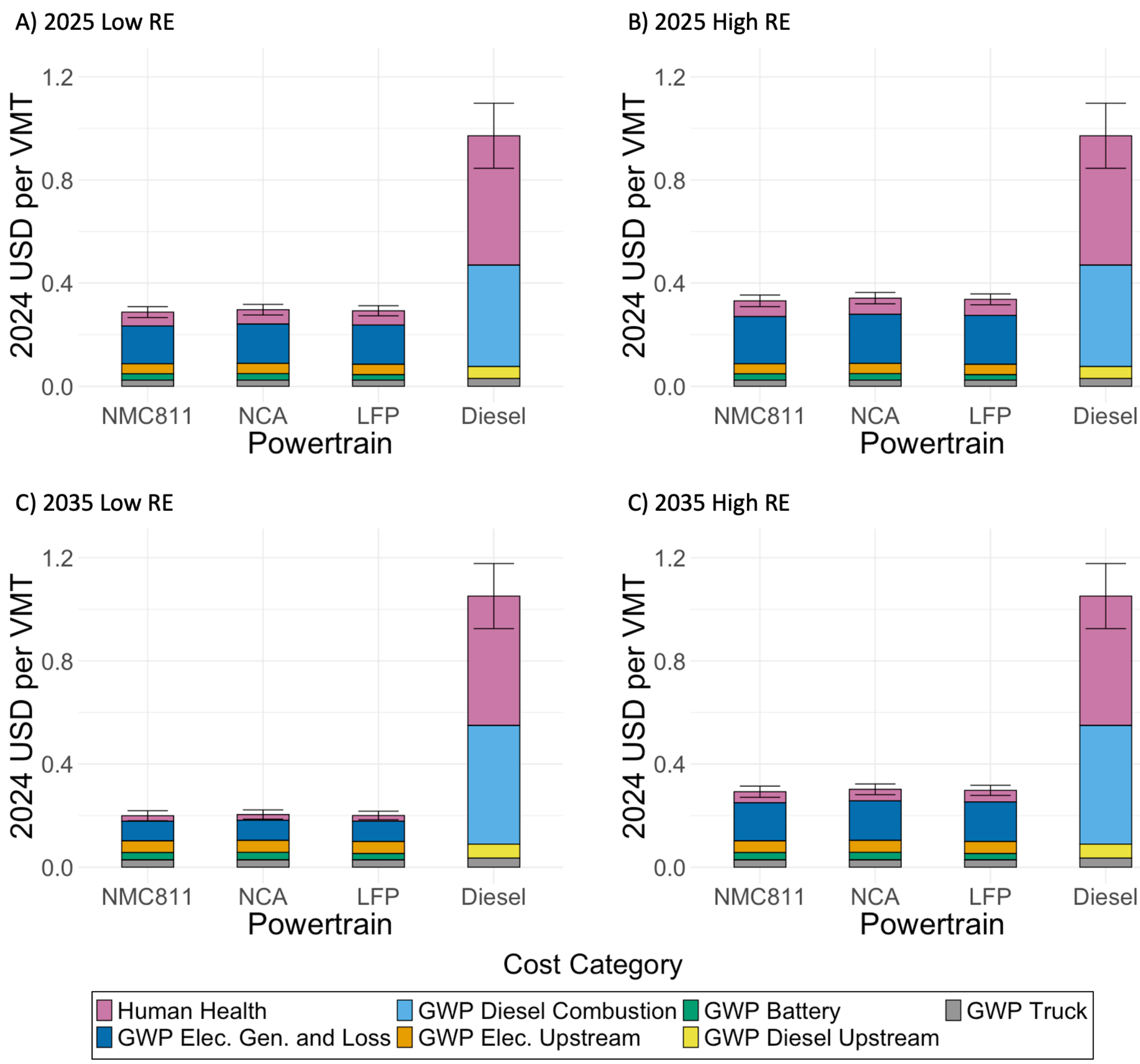

Fig. S17. External costs of BE-HDVs and diesel HDVs performing long-haul freight in varying years and renewable energy cost scenarios with a 2% discount rate for human health impacts, a 2% discount rate for GWP, and a SCC of \$212/tonne CO_{2eq} and \$248/tonne CO_{2eq} for 2025 and 2035 respectively. (**A**) Visualized external costs in 2025 under a low renewable energy cost scenario. (**B**) Visualized external costs in 2025 under a high renewable energy cost scenario. (**C**) Visualized external costs in 2035 under a low renewable energy cost scenario. (**D**) Visualized external costs in 2035 under a high renewable energy cost scenario.

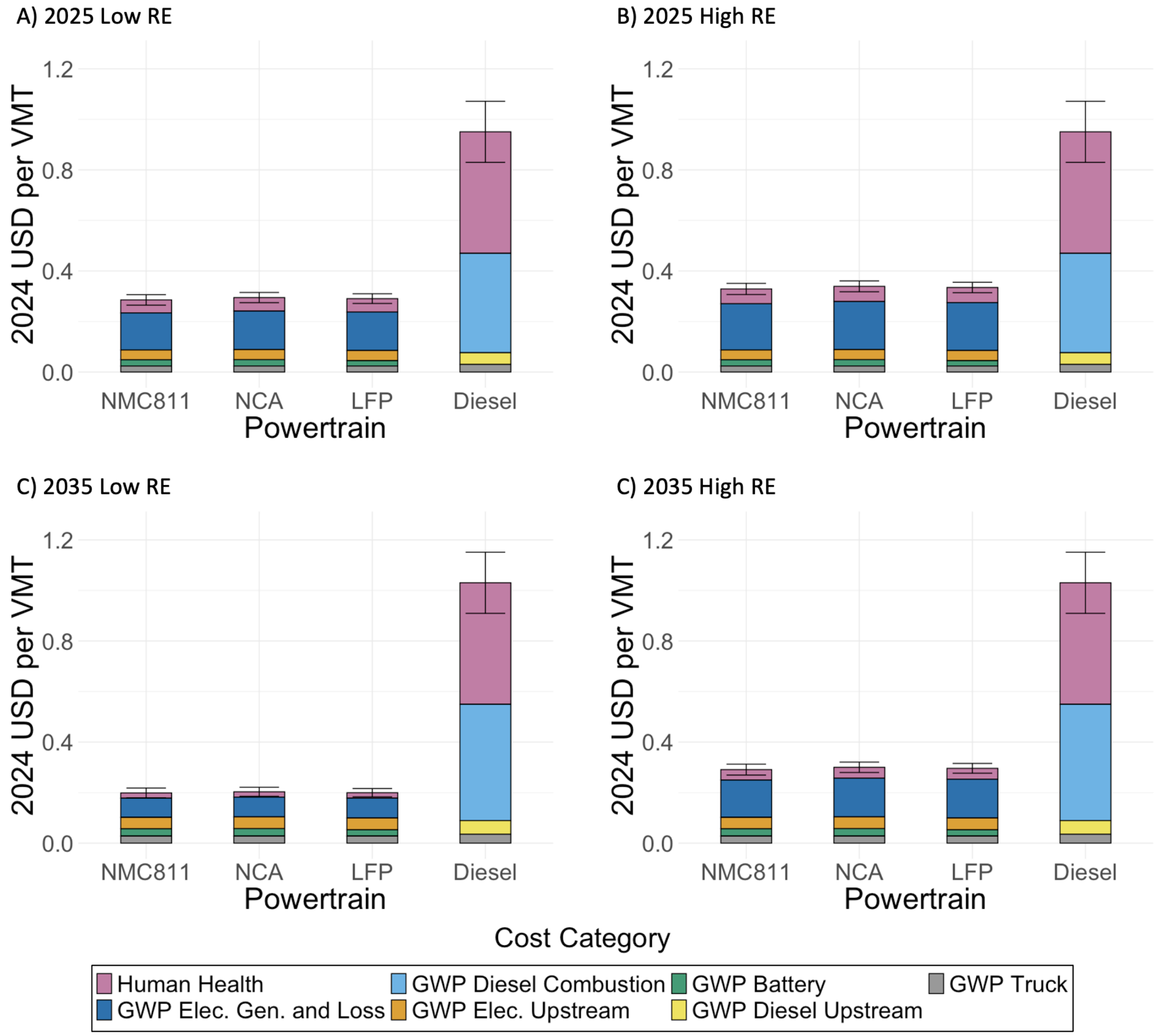

Fig. S18. External costs of BE-HDVs and diesel HDVs performing long-haul freight in varying years and renewable energy cost scenarios with a 5% discount rate for human health impacts, a 2% discount rate for GWP, and a SCC of \$212/tonne CO_{2eq} and \$248/tonne CO_{2eq} for 2025 and 2035 respectively. (**A**) Visualized external costs in 2025 under a low renewable energy cost scenario. (**B**) Visualized external costs in 2025 under a high renewable energy cost scenario. (**C**) Visualized external costs in 2035 under a low renewable energy cost scenario. (**D**) Visualized external costs in 2035 under a high renewable energy cost scenario.

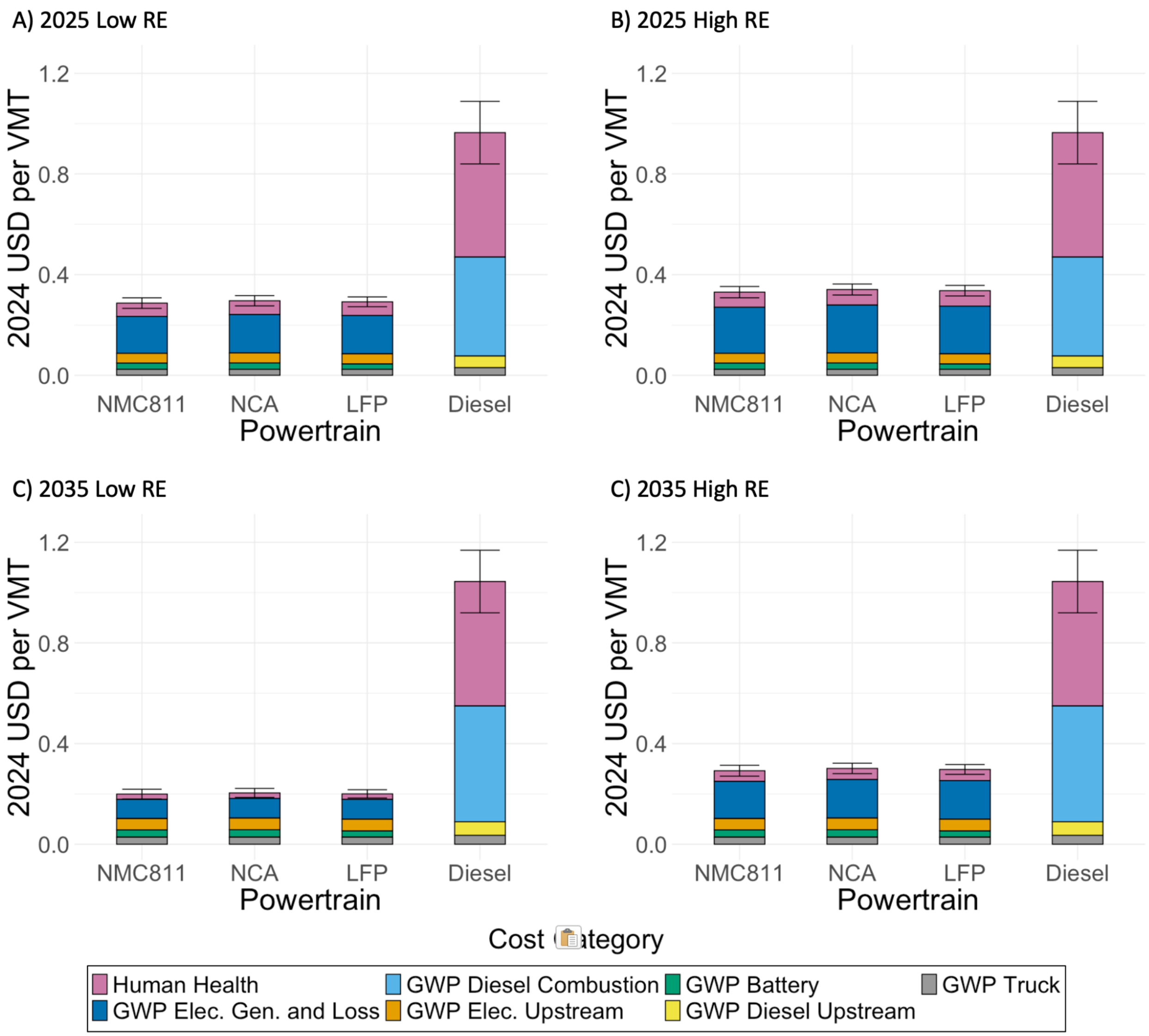

Fig S19. External costs of BE-HDVs and diesel HDVs performing long-haul freight in varying years and renewable energy cost scenarios with a 3% discount rate for human health impacts, a 2% discount rate for GWP, and a SCC of $212/tonne CO_{2eq} and $248/tonne CO_{2eq} for 2025 and 2035 respectively. a) Visualized social costs in 2025 under a low renewable energy cost scenario. b) Visualized social costs in 2025 under a high renewable energy cost scenario. c) Visualized social costs in 2035 under a low renewable energy cost scenario. d) Visualized social costs in 2035 under a high renewable energy cost scenario.

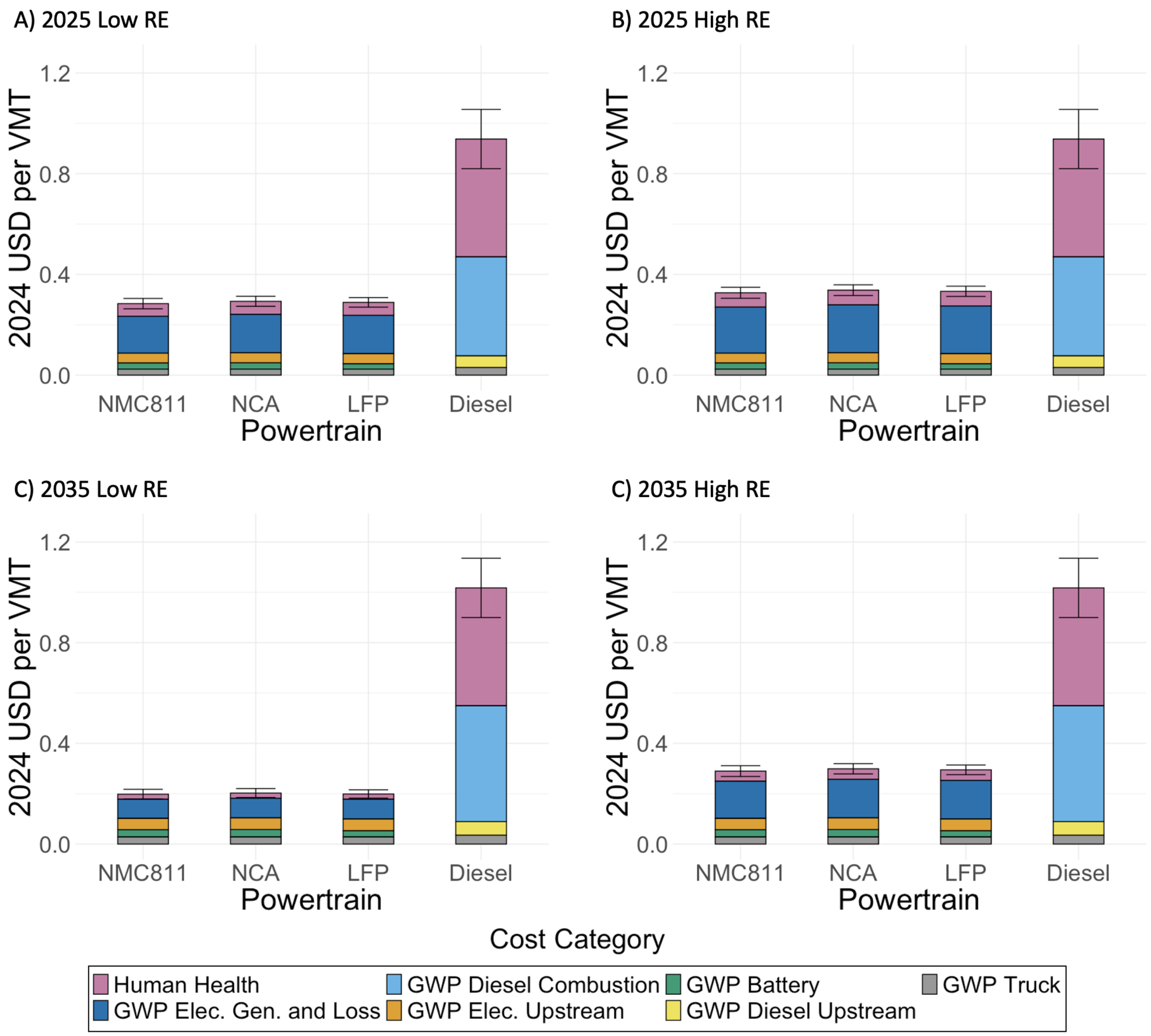

Fig S20. External costs of BE-HDVs and diesel HDVs performing long-haul freight in varying years and renewable energy cost scenarios with a 7% discount rate for human health impacts, a 2% discount rate for GWP, and a SCC of \$212/tonne CO_{2eq} and \$248/tonne CO_{2eq} for 2025 and 2035 respectively. a) Visualized social costs in 2025 under a low renewable energy cost scenario. b) Visualized social costs in 2025 under a high renewable energy cost scenario. c) Visualized social costs in 2035 under a low renewable energy cost scenario. d) Visualized social costs in 2035 under a high renewable energy cost scenario.

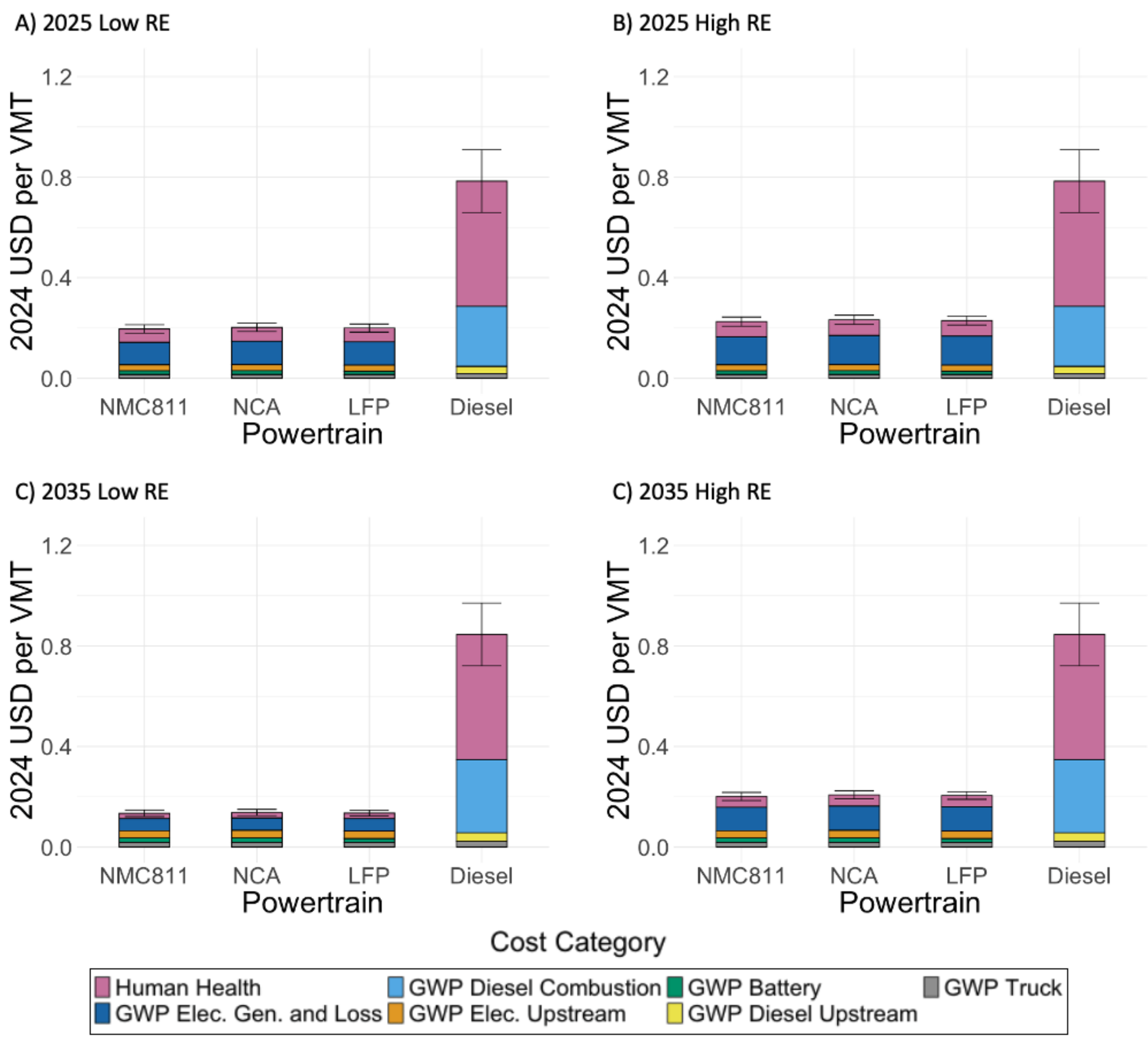

Fig S21. External costs of BE-HDVs and diesel HDVs performing long-haul freight in varying years and renewable energy cost scenarios with a 2.5% discount rate for human health impacts, a 2.5% discount rate for GWP, and a SCC of \$130/tonne CO_{2eq} and \$158/tonne CO_{2eq} for 2025 and 2035 respectively. a) Visualized social costs in 2025 under a low renewable energy cost scenario. b) Visualized social costs in 2025 under a high renewable energy cost scenario. c) Visualized social costs in 2035 under a low renewable energy cost scenario. d) Visualized social costs in 2035 under a high renewable energy cost scenario.

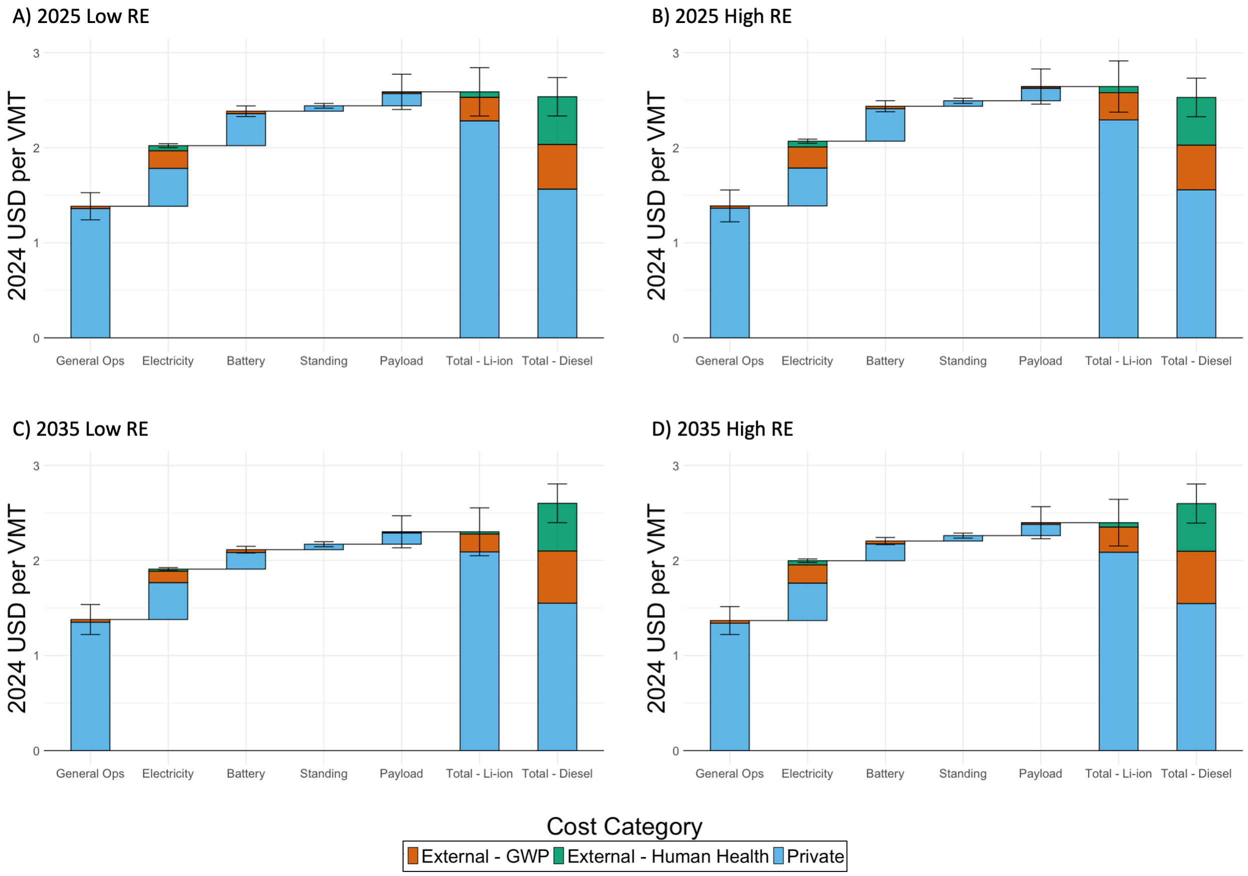

Fig S22. **Social costs of NMC811 BE-HDVs and diesel HDVs performing long-haul freight in varying years and renewable energy cost scenarios with a 2% discount rate for human health and private impacts, a 2% discount rate for GWP impacts, and a SCC of \$212/tonne CO_{2eq} and \$248/tonne CO_{2eq} for 2025 and 2035 respectively.** (**A**) Visualized social costs in 2025 under a low renewable energy cost scenario. (**B**) Visualized social costs in 2025 under a high renewable energy cost scenario. (**C**) Visualized social costs in 2035 under a low renewable energy cost scenario. (**D**) Visualized social costs in 2035 under a high renewable energy cost scenario.

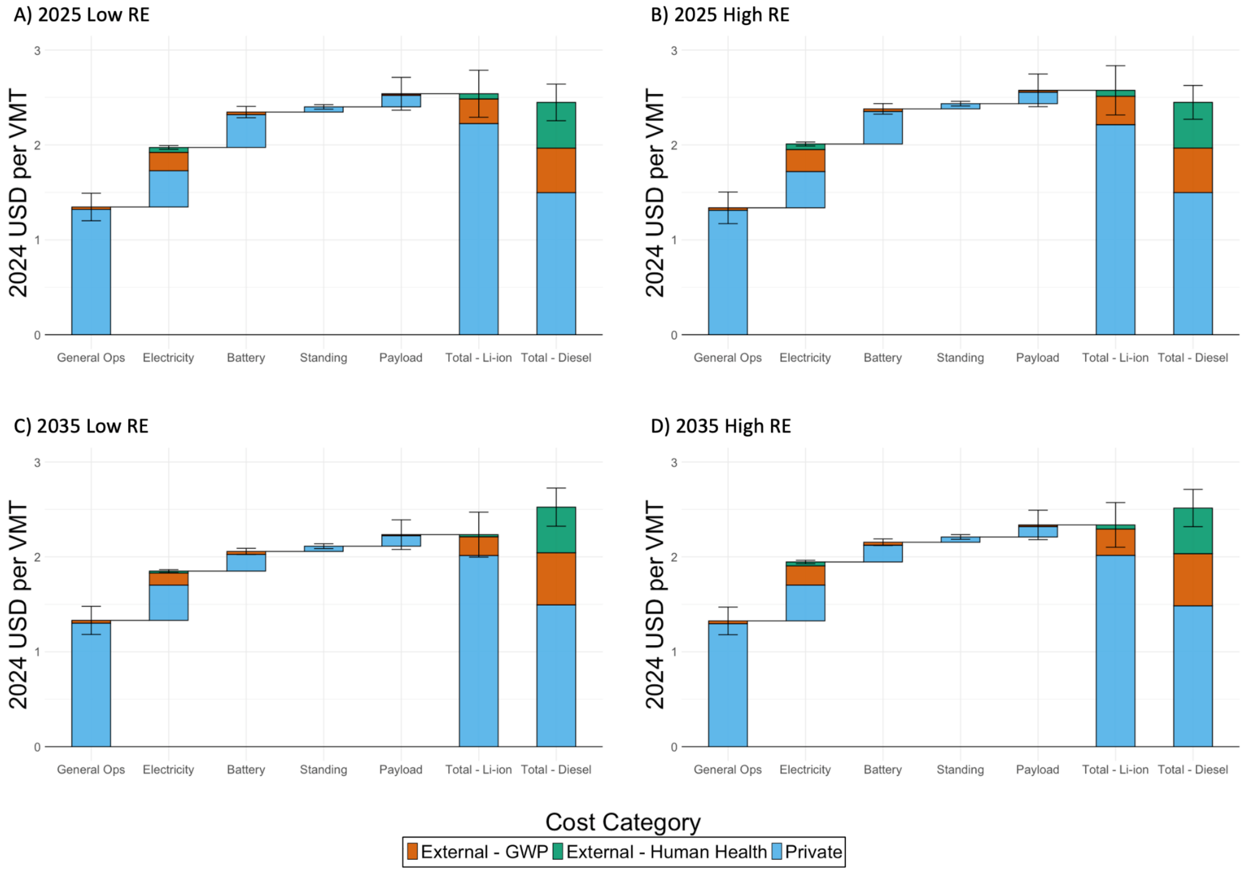

Fig S23. **Social costs of NMC811 BE-HDVs and diesel HDVs performing long-haul freight in varying years and renewable energy cost scenarios with a 5% discount rate for human health and private impacts, a 2% discount rate for GWP impacts, and a SCC of \$212/tonne CO_{2eq} and \$248/tonne CO_{2eq} for 2025 and 2035 respectively.** (**A**) Visualized social costs in 2025 under a low renewable energy cost scenario. (**B**) Visualized social costs in 2025 under a high renewable energy cost scenario. (**C**) Visualized social costs in 2035 under a low renewable energy cost scenario. (**D**) Visualized social costs in 2035 under a high renewable energy cost scenario.

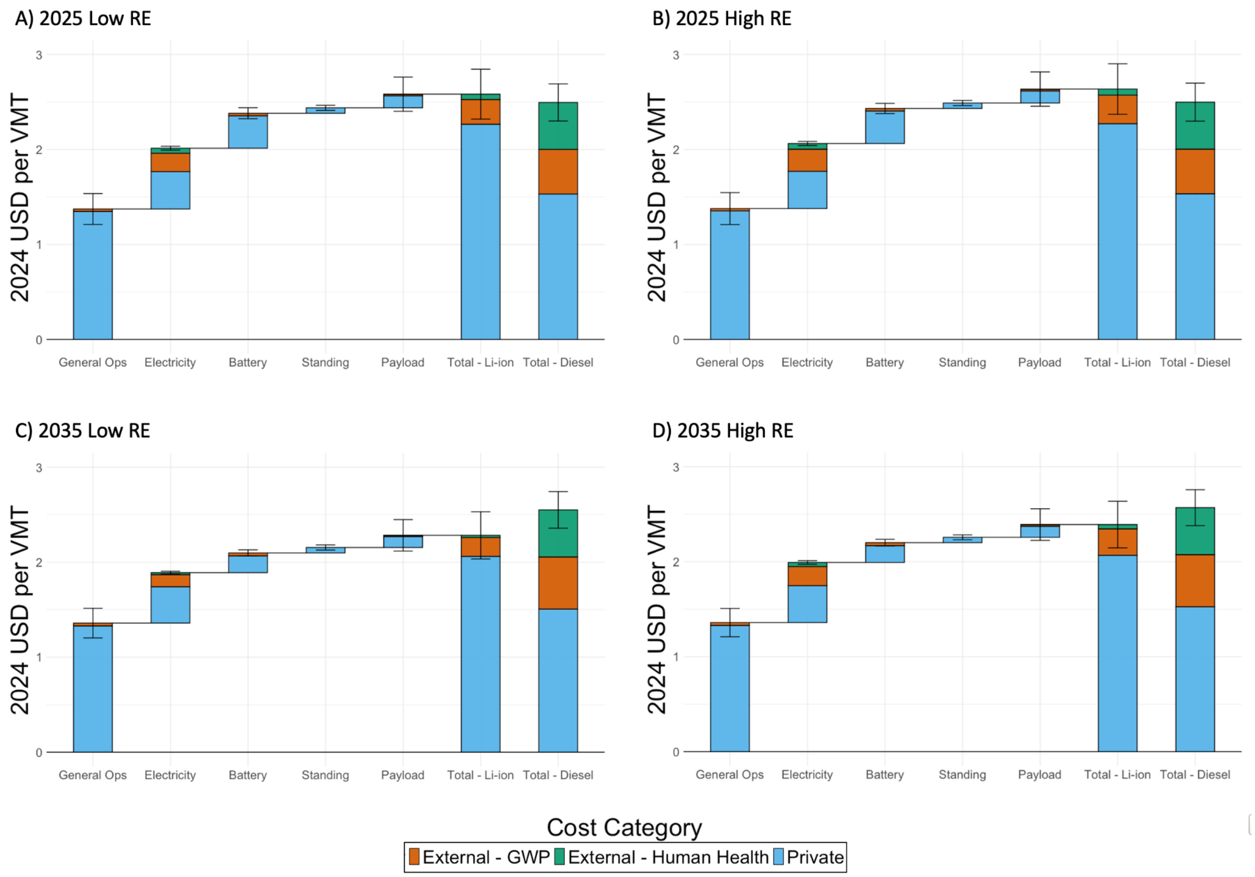

Fig S24. Social costs of NMC811 BE-HDVs and diesel HDVs performing long-haul freight in varying years and renewable energy cost scenarios with a 3% discount rate for human health and private impacts, a 2% discount rate for GWP impacts, and a SCC of $212/tonne CO_{2eq} and $248/tonne CO_{2eq} for 2025 and 2035 respectively. (**A**) Visualized social costs in 2025 under a low renewable energy cost scenario. (**B**) Visualized social costs in 2025 under a high renewable energy cost scenario. (**C**) Visualized social costs in 2035 under a low renewable energy cost scenario. (**D**) Visualized social costs in 2035 under a high renewable energy cost scenario.

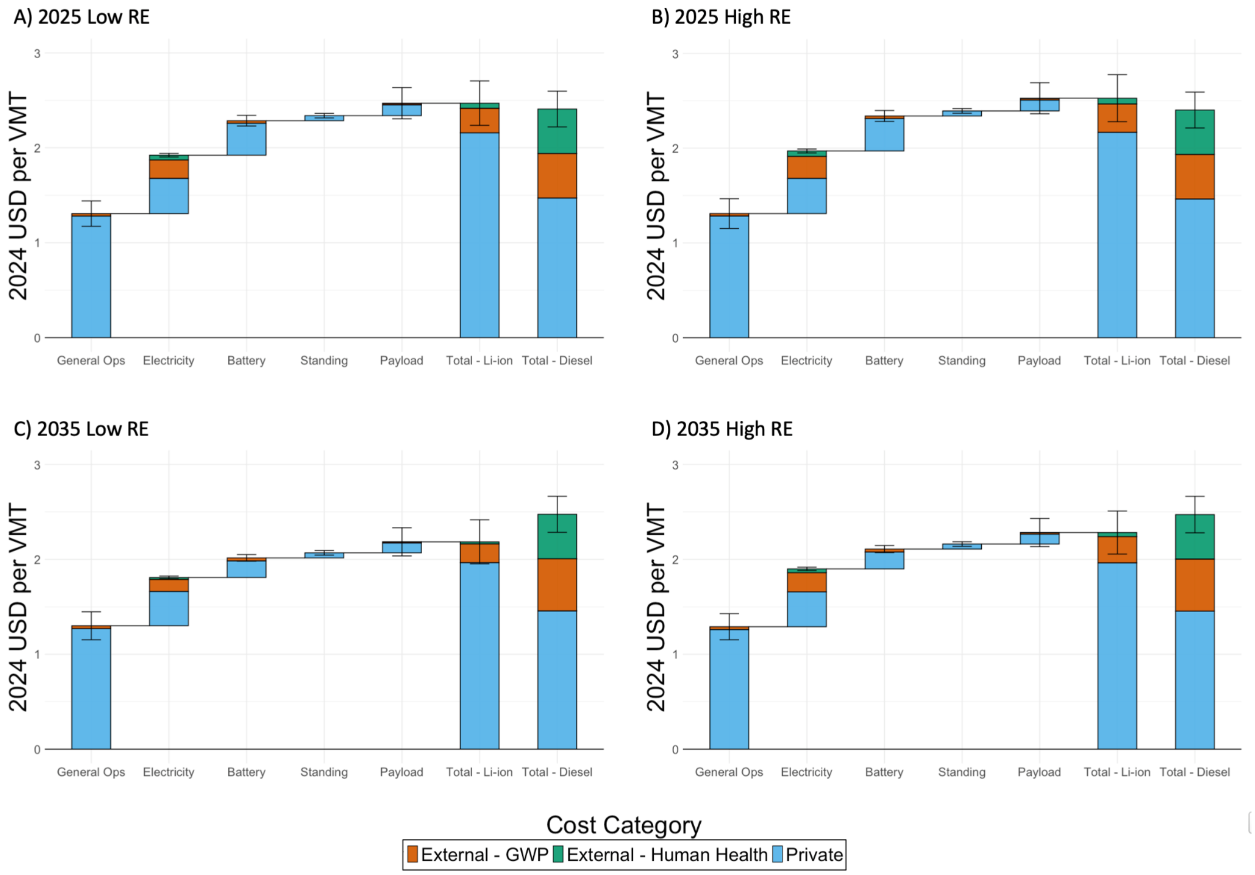

Fig S25. **Social costs of NMC811 BE-HDVs and diesel HDVs performing long-haul freight in varying years and renewable energy cost scenarios with a 7% discount rate for human health and private impacts, a 2% discount rate for GWP impacts, and a SCC of $212/tonne CO_{2eq} and $248/tonne CO_{2eq} for 2025 and 2035 respectively.** (**A**) Visualized social costs in 2025 under a low renewable energy cost scenario. (**B**) Visualized social costs in 2025 under a high renewable energy cost scenario. (**C**) Visualized social costs in 2035 under a low renewable energy cost scenario. (**D**) Visualized social costs in 2035 under a high renewable energy cost scenario.

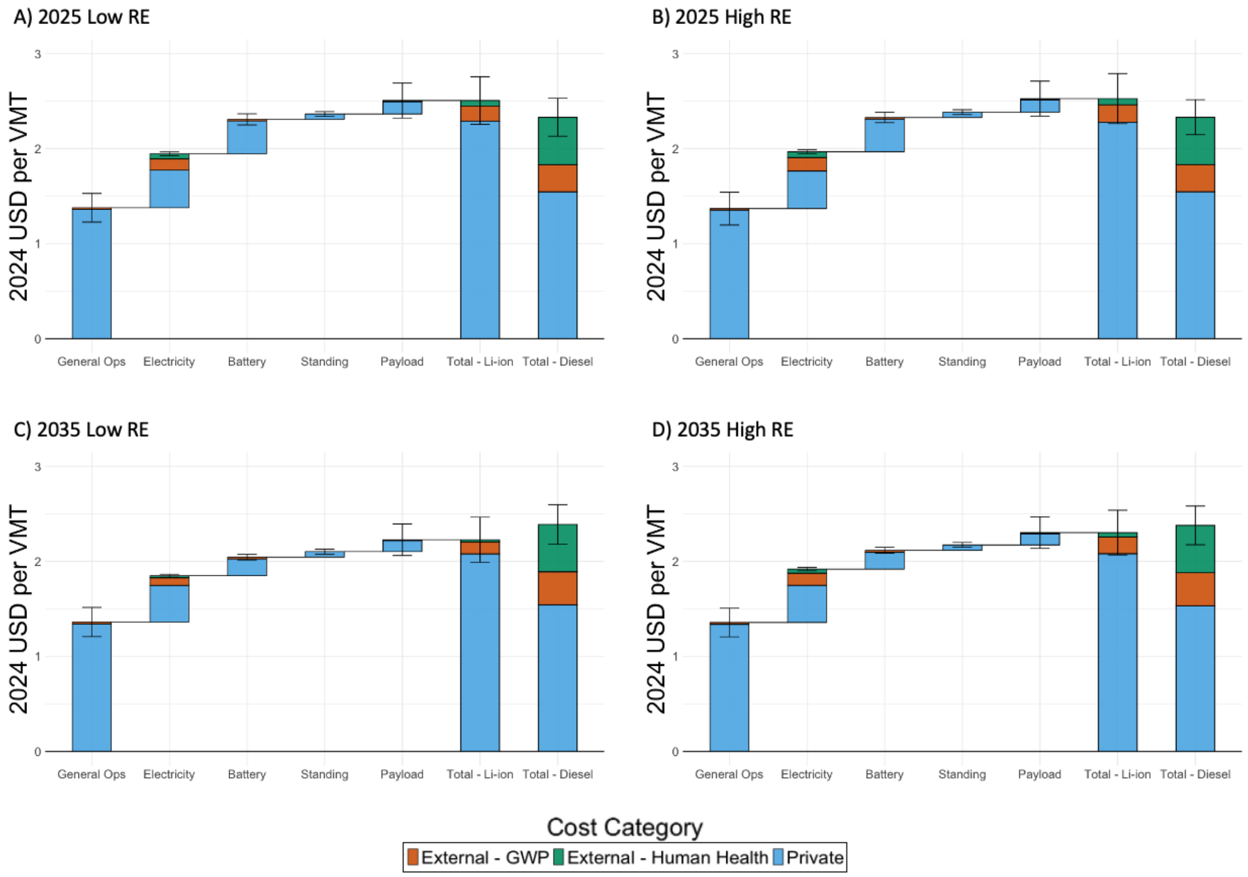

Fig S26. **Social costs of NMC811 BE-HDVs and diesel HDVs performing long-haul freight in varying years and renewable energy cost scenarios with a 2.5% discount rate for human health and private impacts, a 2.5% discount rate for GWP impacts, and a SCC of \$130/tonne CO_{2eq} and \$158/tonne CO_{2eq} for 2025 and 2035 respectively.** (**A**) Visualized social costs in 2025 under a low renewable energy cost scenario. (**B**) Visualized social costs in 2025 under a high renewable energy cost scenario. (**C**) Visualized social costs in 2035 under a low renewable energy cost scenario. (**D**) Visualized social costs in 2035 under a high renewable energy cost scenario.

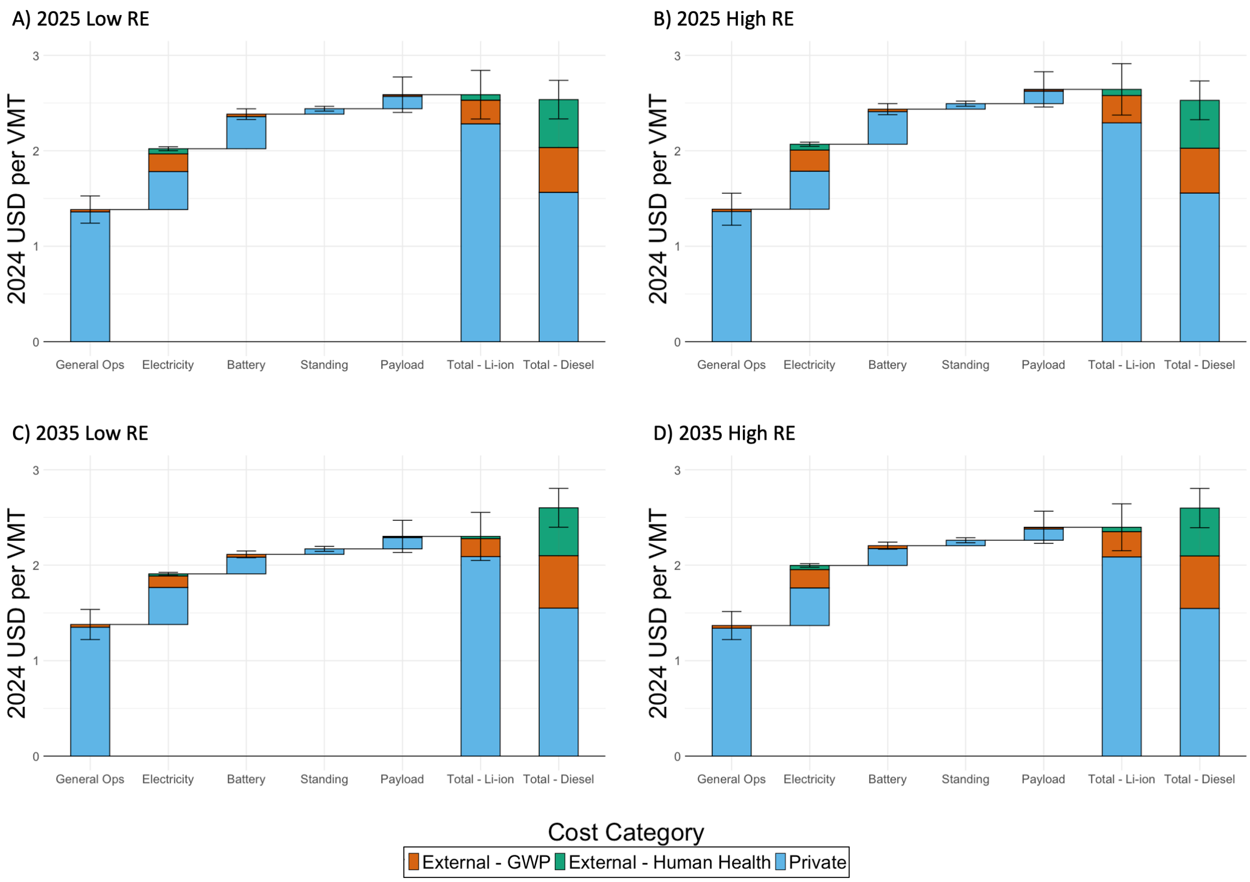

Fig S27. **Social costs of NCA BE-HDVs and diesel HDVs performing long-haul freight in varying years and renewable energy cost scenarios with a 2% discount rate for human health and private impacts, a 2% discount rate for GWP impacts, and a SCC of \$212/tonne CO_{2eq} and \$248/tonne CO_{2eq} for 2025 and 2035 respectively.** (**A**) Visualized social costs in 2025 under a low renewable energy cost scenario. (**B**) Visualized social costs in 2025 under a high renewable energy cost scenario. (**C**) Visualized social costs in 2035 under a low renewable energy cost scenario. (**D**) Visualized social costs in 2035 under a high renewable energy cost scenario.

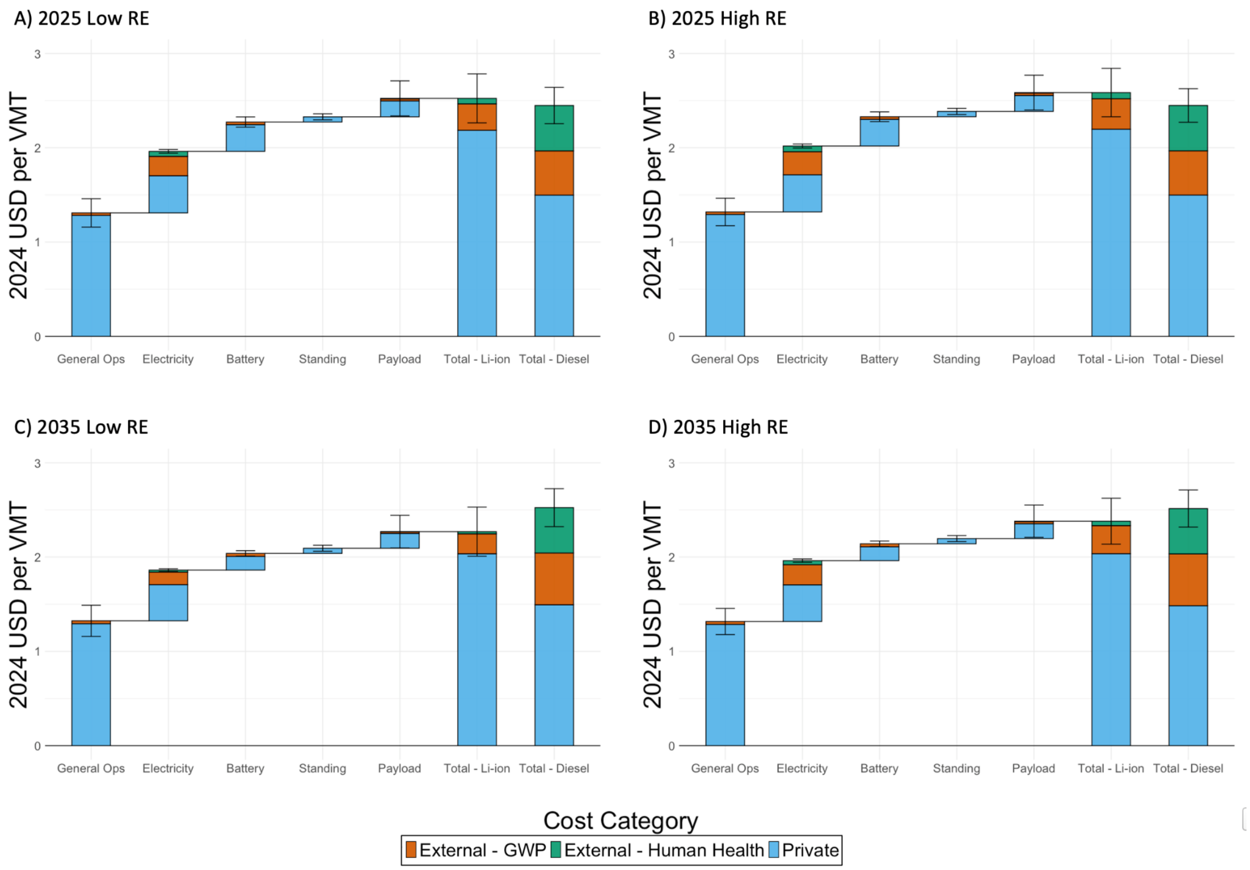

Fig S28. **Social costs of NCA BE-HDVs and diesel HDVs performing long-haul freight in varying years and renewable energy cost scenarios with a 5% discount rate for human health and private impacts, a 2% discount rate for GWP impacts, and a SCC of $212/tonne CO_{2eq} and $248/tonne CO_{2eq} for 2025 and 2035 respectively.** (**A**) Visualized social costs in 2025 under a low renewable energy cost scenario. (**B**) Visualized social costs in 2025 under a high renewable energy cost scenario. (**C**) Visualized social costs in 2035 under a low renewable energy cost scenario. (**D**) Visualized social costs in 2035 under a high renewable energy cost scenario.

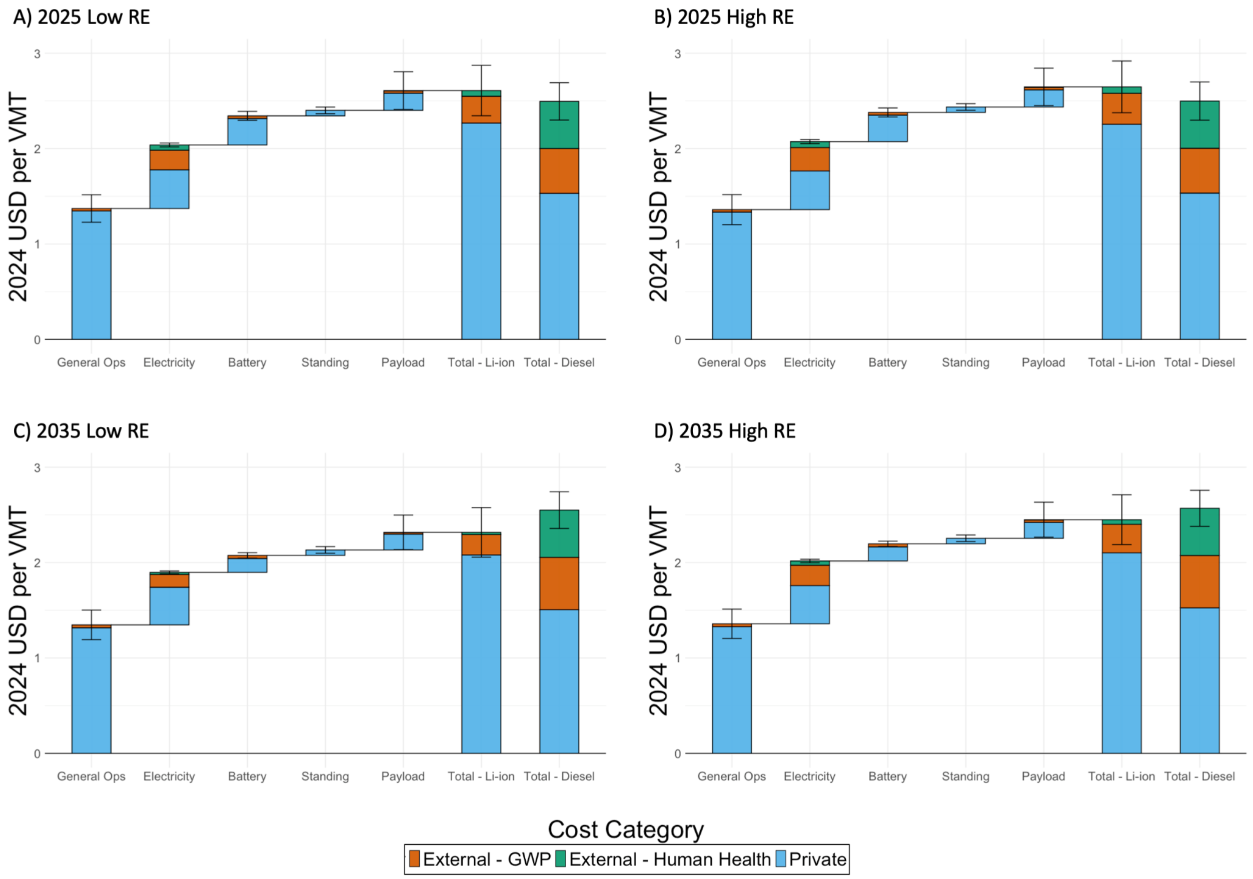

Fig S29. Social costs of NCA BE-HDVs and diesel HDVs performing long-haul freight in varying years and renewable energy cost scenarios with a 3% discount rate for human health and private impacts, a 2% discount rate for GWP impacts, and a SCC of $212/tonne CO_{2eq} and $248/tonne CO_{2eq} for 2025 and 2035 respectively. (**A**) Visualized social costs in 2025 under a low renewable energy cost scenario. (**B**) Visualized social costs in 2025 under a high renewable energy cost scenario. (**C**) Visualized social costs in 2035 under a low renewable energy cost scenario. (**D**) Visualized social costs in 2035 under a high renewable energy cost scenario.

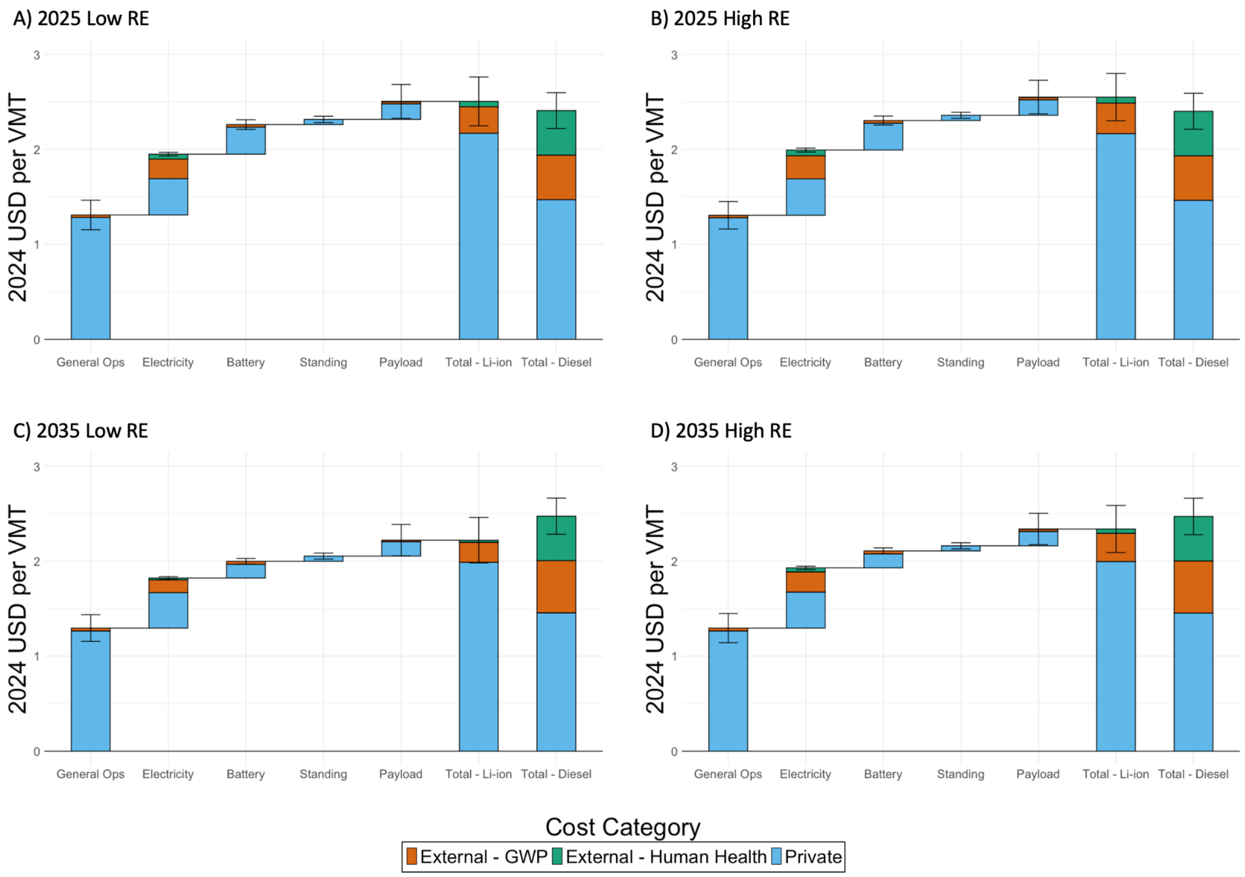

Fig S30. **Social costs of NCA BE-HDVs and diesel HDVs performing long-haul freight in varying years and renewable energy cost scenarios with a 7% discount rate for human health and private impacts, a 2% discount rate for GWP impacts, and a SCC of $212/tonne CO_{2eq} and $248/tonne CO_{2eq} for 2025 and 2035 respectively.** (**A**) Visualized social costs in 2025 under a low renewable energy cost scenario. (**B**) Visualized social costs in 2025 under a high renewable energy cost scenario. (**C**) Visualized social costs in 2035 under a low renewable energy cost scenario. (**D**) Visualized social costs in 2035 under a high renewable energy cost scenario.

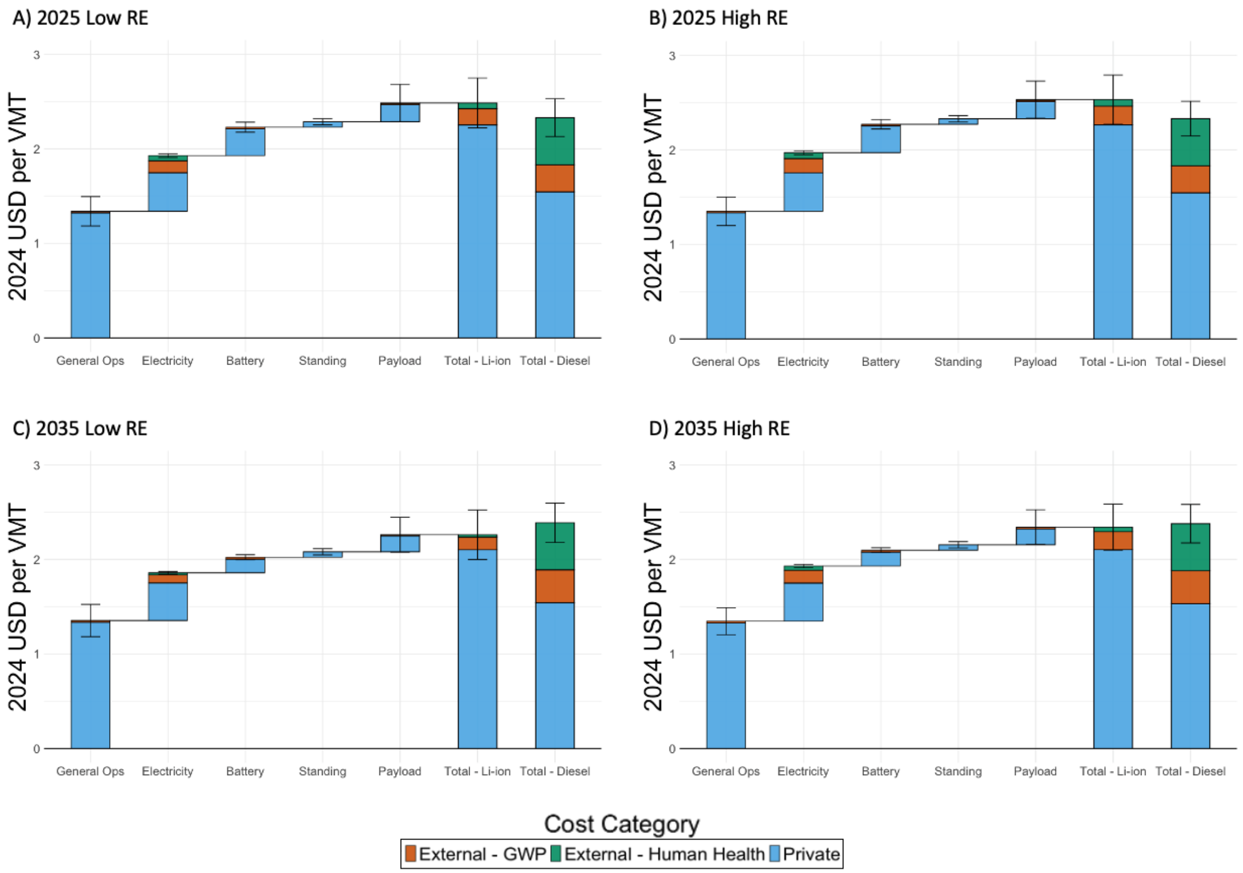

Fig S31. **Social costs of NCA BE-HDVs and diesel HDVs performing long-haul freight in varying years and renewable energy cost scenarios with a 2.5% discount rate for human health and private impacts, a 2.5% discount rate for GWP impacts, and a SCC of \$130/tonne CO_{2eq} and \$158/tonne CO_{2eq} for 2025 and 2035 respectively.** (**A**) Visualized social costs in 2025 under a low renewable energy cost scenario. (**B**) Visualized social costs in 2025 under a high renewable energy cost scenario. (**C**) Visualized social costs in 2035 under a low renewable energy cost scenario. (**D**) Visualized social costs in 2035 under a high renewable energy cost scenario.

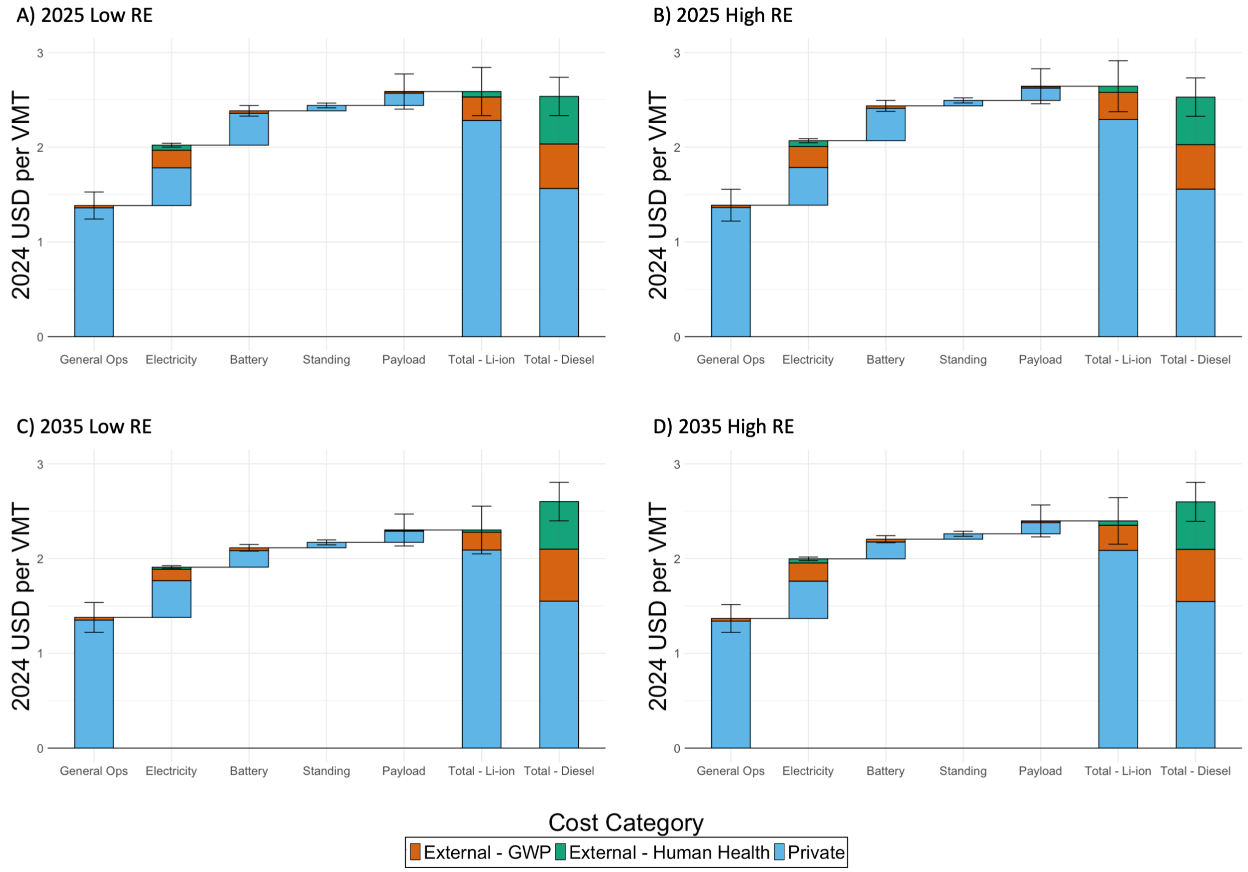

Fig S32. Social costs of LFP BE-HDVs and diesel HDVs performing long-haul freight in varying years and renewable energy cost scenarios with a 2% discount rate for human health and private impacts, a 2% discount rate for GWP impacts, and a SCC of \$212/tonne CO_{2eq} and \$248/tonne CO_{2eq} for 2025 and 2035 respectively. (**A**) Visualized social costs in 2025 under a low renewable energy cost scenario. (**B**) Visualized social costs in 2025 under a high renewable energy cost scenario. (**C**) Visualized social costs in 2035 under a low renewable energy cost scenario. (**D**) Visualized social costs in 2035 under a high renewable energy cost scenario.

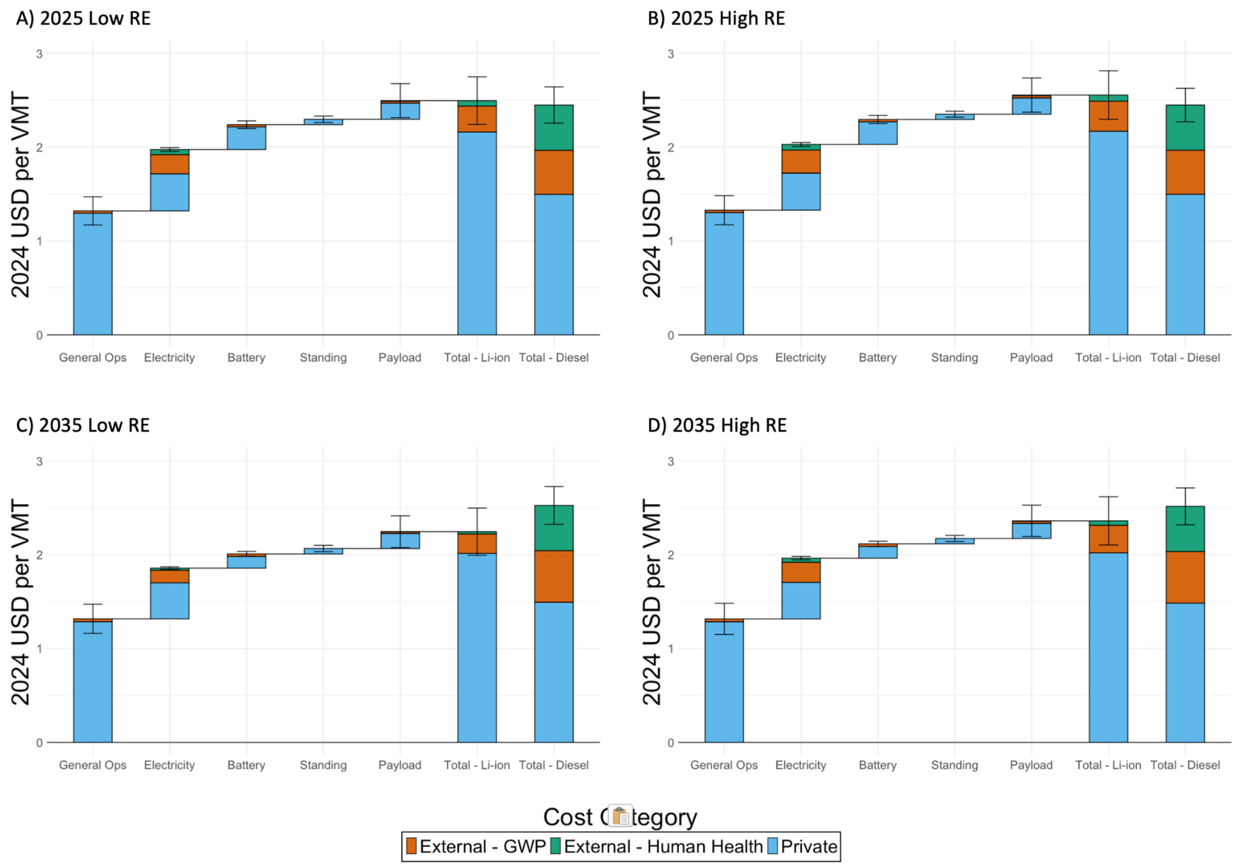

Fig S33. Social costs of LFP BE-HDVs and diesel HDVs performing long-haul freight in varying years and renewable energy cost scenarios with a 5% discount rate for human health and private impacts, a 2% discount rate for GWP impacts, and a SCC of $212/tonne CO_{2eq} and $248/tonne CO_{2eq} for 2025 and 2035 respectively. (**A**) Visualized social costs in 2025 under a low renewable energy cost scenario. (**B**) Visualized social costs in 2025 under a high renewable energy cost scenario. (**C**) Visualized social costs in 2035 under a low renewable energy cost scenario. (**D**) Visualized social costs in 2035 under a high renewable energy cost scenario.

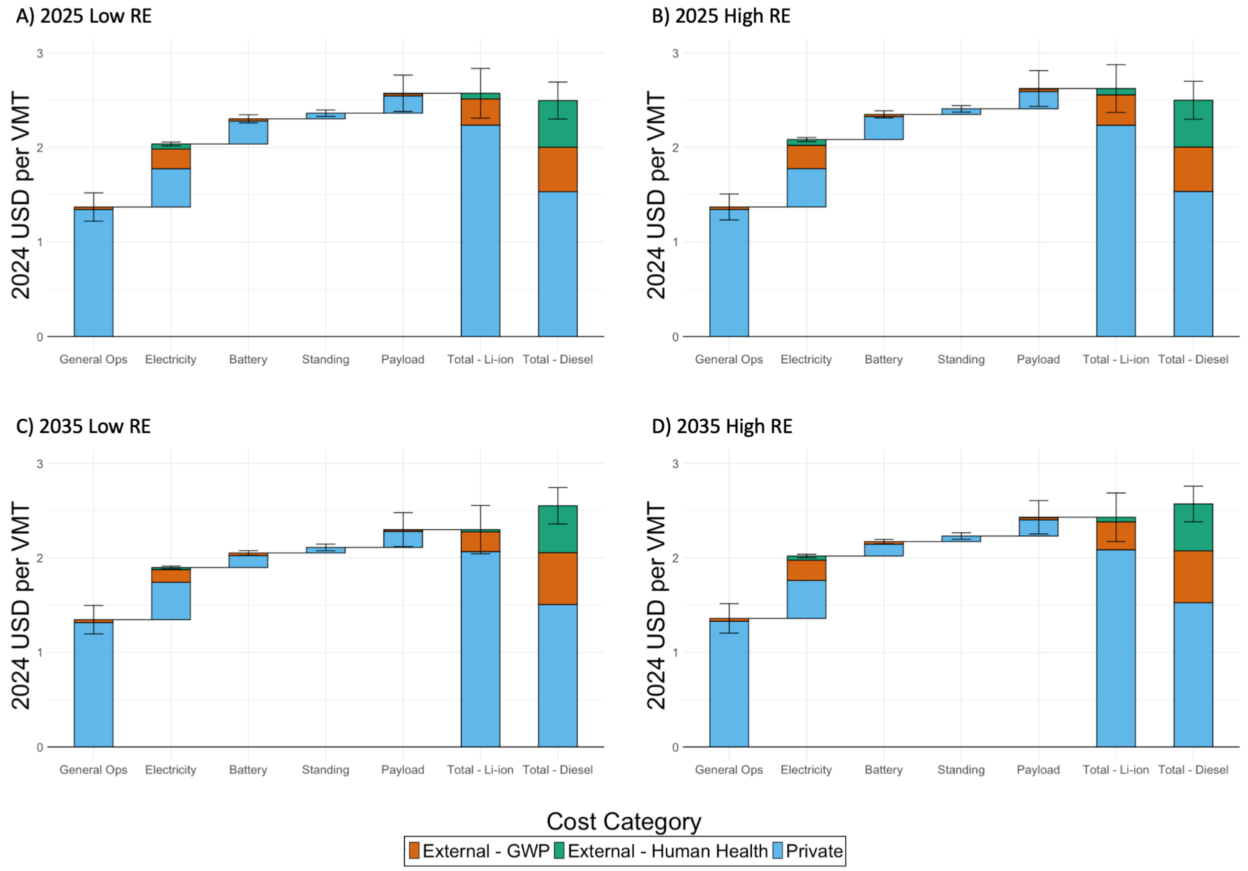

Fig S34. **Social costs of LFP BE-HDVs and diesel HDVs performing long-haul freight in varying years and renewable energy cost scenarios with a 3% discount rate for human health and private impacts, a 2% discount rate for GWP impacts, and a SCC of \$212/tonne CO_{2eq} and \$248/tonne CO_{2eq} for 2025 and 2035 respectively.** (**A**) Visualized social costs in 2025 under a low renewable energy cost scenario. (**B**) Visualized social costs in 2025 under a high renewable energy cost scenario. (**C**) Visualized social costs in 2035 under a low renewable energy cost scenario. (**D**) Visualized social costs in 2035 under a high renewable energy cost scenario.

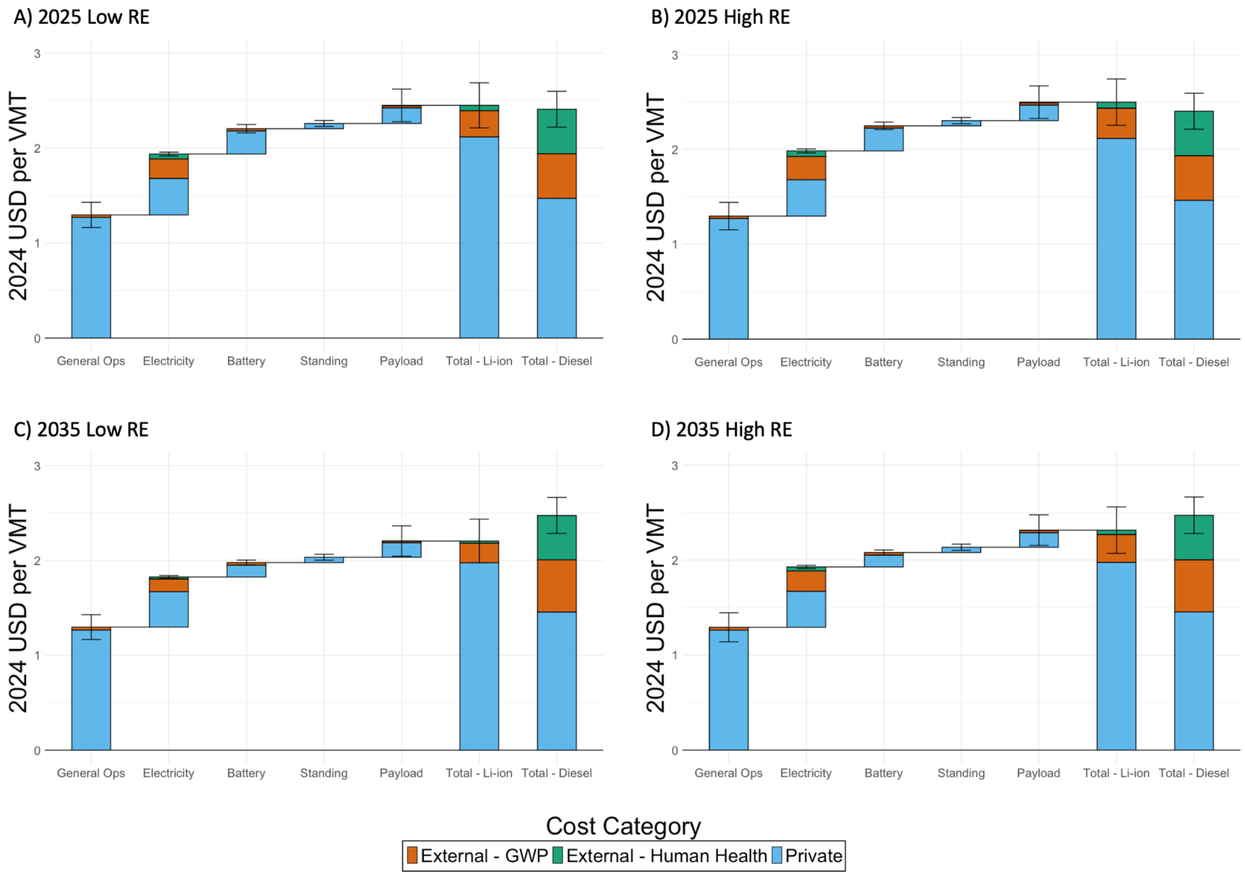

Fig S35. **Social costs of LFP BE-HDVs and diesel HDVs performing long-haul freight in varying years and renewable energy cost scenarios with a 7% discount rate for human health and private impacts, a 2% discount rate for GWP impacts, and a SCC of $212/tonne CO_{2eq} and $248/tonne CO_{2eq} for 2025 and 2035 respectively.** (**A**) Visualized social costs in 2025 under a low renewable energy cost scenario. (**B**) Visualized social costs in 2025 under a high renewable energy cost scenario. (**C**) Visualized social costs in 2035 under a low renewable energy cost scenario. (**D**) Visualized social costs in 2035 under a high renewable energy cost scenario.

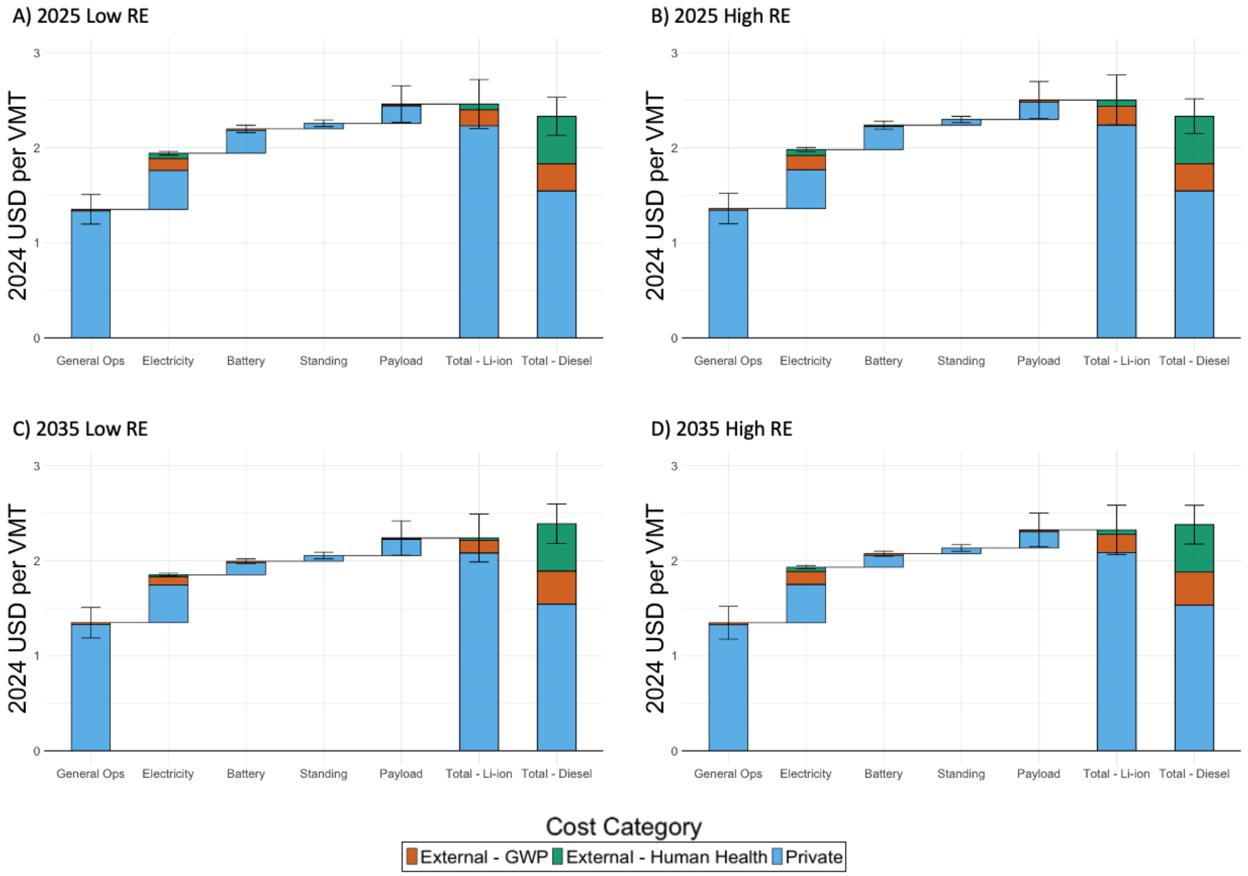

Fig S36. Social costs of LFP BE-HDVs and diesel HDVs performing long-haul freight in varying years and renewable energy cost scenarios with a 2.5% discount rate for human health and private impacts, a 2.5% discount rate for GWP impacts, and a SCC of \$130/tonne CO_{2eq} and \$158/tonne CO_{2eq} for 2025 and 2035 respectively. (**A**) Visualized social costs in 2025 under a low renewable energy cost scenario. (**B**) Visualized social costs in 2025 under a high renewable energy cost scenario. (**C**) Visualized social costs in 2035 under a low renewable energy cost scenario. (**D**) Visualized social costs in 2035 under a high renewable energy cost scenario.

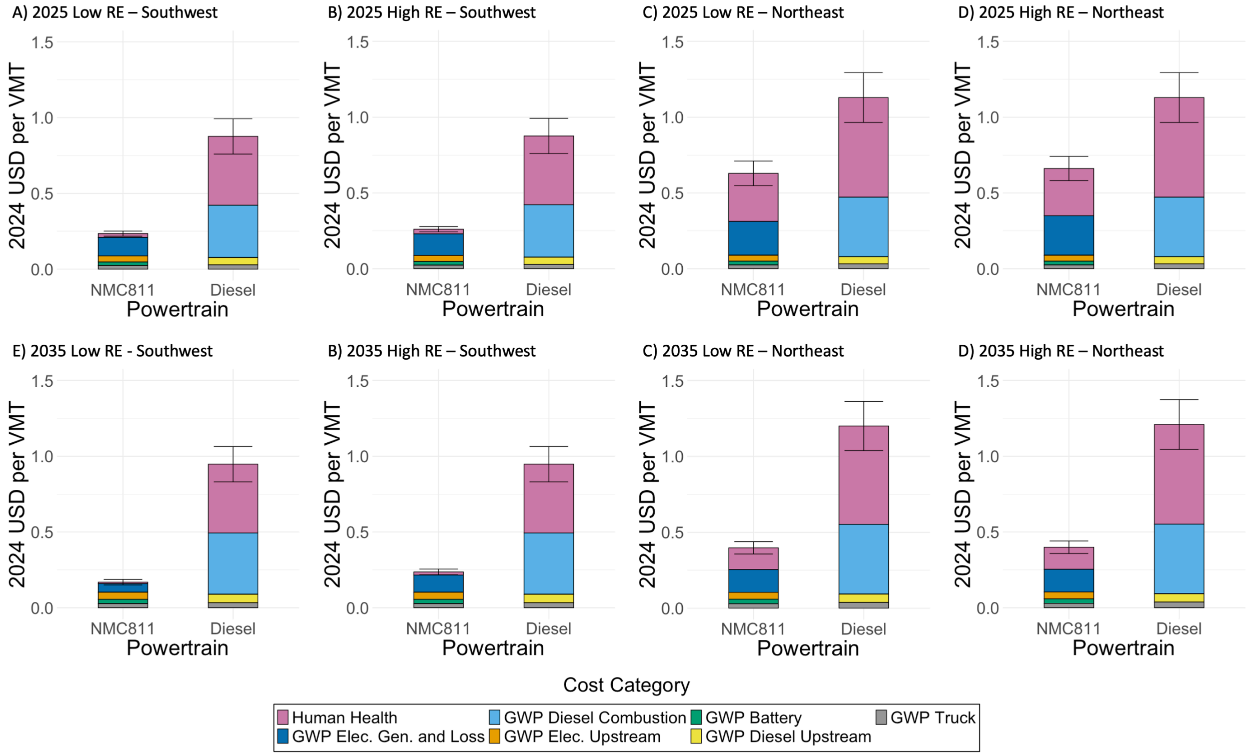

Fig S37. External costs of NMC811 BE-HDVs and diesel HDVs performing long-haul freight within different highway corridors with a 2% discount rate. a) Visualized social costs on the southwest corridor in 2024 under a low renewable energy cost scenario. b) Visualized social costs on the southwest corridor in 2024 under a high renewable energy cost scenario. c) Visualized social costs on the southwest corridor in 2035 under a low renewable energy cost scenario. d) Visualized social costs on the southwest corridor in 2024 under a low renewable energy cost scenario. e) Visualized social costs on the northeast corridor in 2024 under a low renewable energy cost scenario. f) Visualized social costs on the northeast corridor in 2024 under a high renewable energy cost scenario. g) Visualized social costs on the northeast corridor in 2035 under a low renewable energy cost scenario. h) Visualized social costs on the northeast corridor in 2024 under a low renewable energy cost scenario.

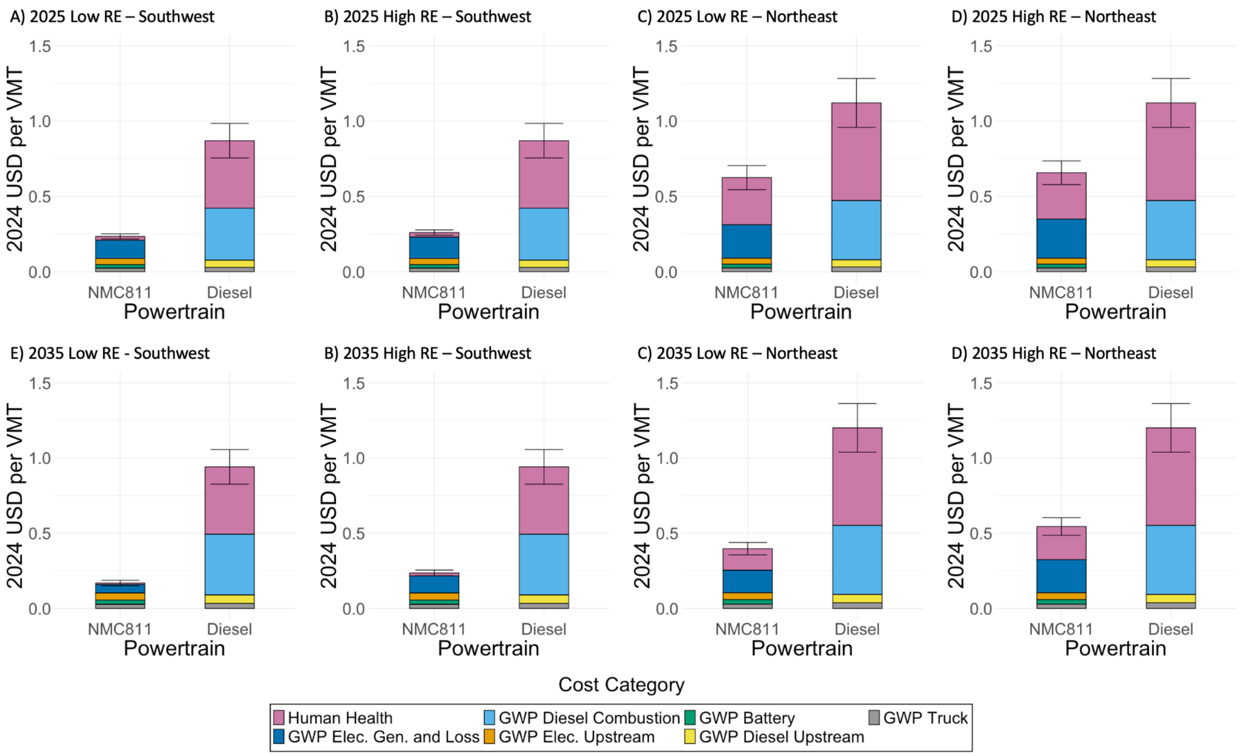

Fig S38. External costs of NMC811 BE-HDVs and diesel HDVs performing long-haul freight within different highway corridors with a 3% discount rate. a) Visualized social costs on the southwest corridor in 2024 under a low renewable energy cost scenario. b) Visualized social costs on the southwest corridor in 2024 under a high renewable energy cost scenario. c) Visualized social costs on the southwest corridor in 2035 under a low renewable energy cost scenario. d) Visualized social costs on the southwest corridor in 2024 under a low renewable energy cost scenario. e) Visualized social costs on the northeast corridor in 2024 under a low renewable energy cost scenario. f) Visualized social costs on the northeast corridor in 2024 under a high renewable energy cost scenario. g) Visualized social costs on the northeast corridor in 2035 under a low renewable energy cost scenario. h) Visualized social costs on the northeast corridor in 2024 under a low renewable energy cost scenario.

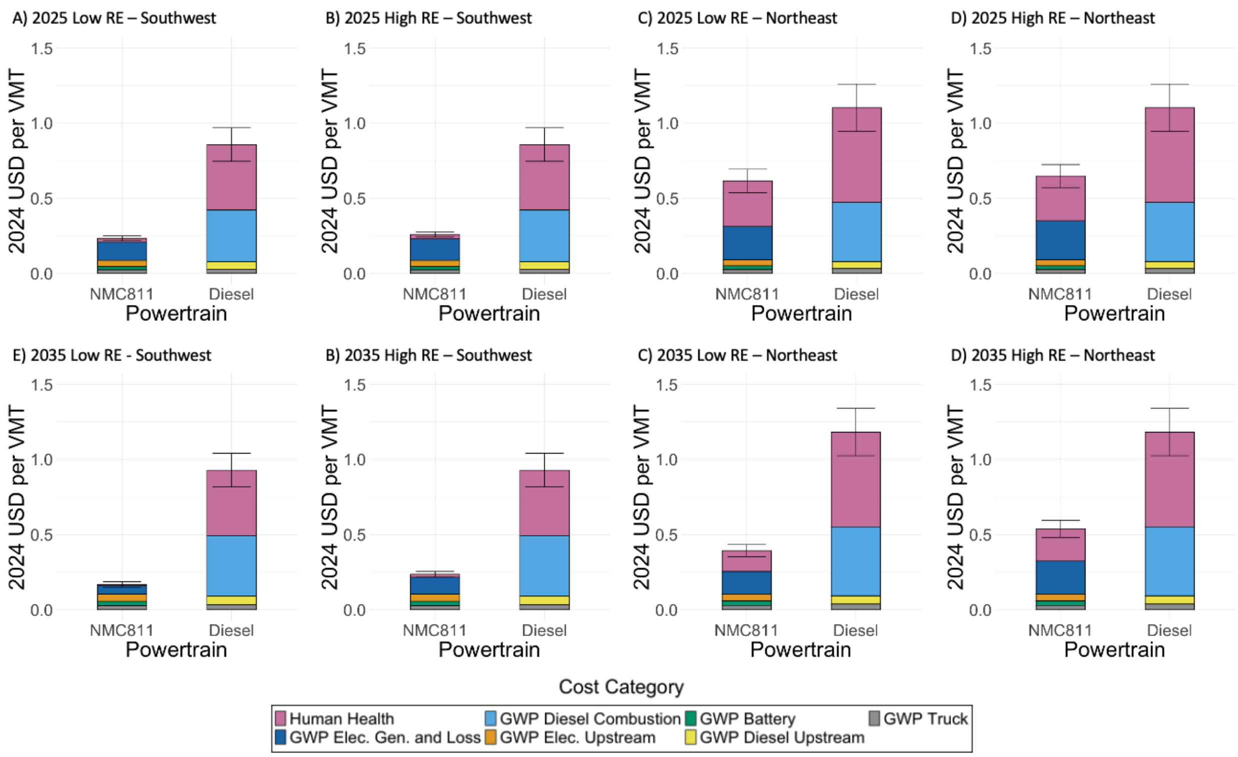

Fig S39. External costs of NMC811 BE-HDVs and diesel HDVs performing long-haul freight within different highway corridors with a 5% discount rate. (**A**) Visualized social costs on the southwest corridor in 2025 under a low renewable energy cost scenario. (**B**) Visualized social costs on the southwest corridor in 2025 under a high renewable energy cost scenario. (**C**) Visualized social costs on the northeast corridor in 2024 under a low renewable energy cost scenario. (**D**) Visualized social costs on the northeast corridor in 2024 under a high renewable energy cost scenario. (**E**) Visualized social costs on the southwest corridor in 2035 under a low renewable energy cost scenario. (**F**) Visualized social costs on the southwest corridor in 2035 under a high renewable energy cost scenario. (**G**) Visualized social costs on the northeast corridor in 2035 under a low renewable energy cost scenario. (**H**) Visualized social costs on the northeast corridor in 2035 under a high renewable energy cost scenario.

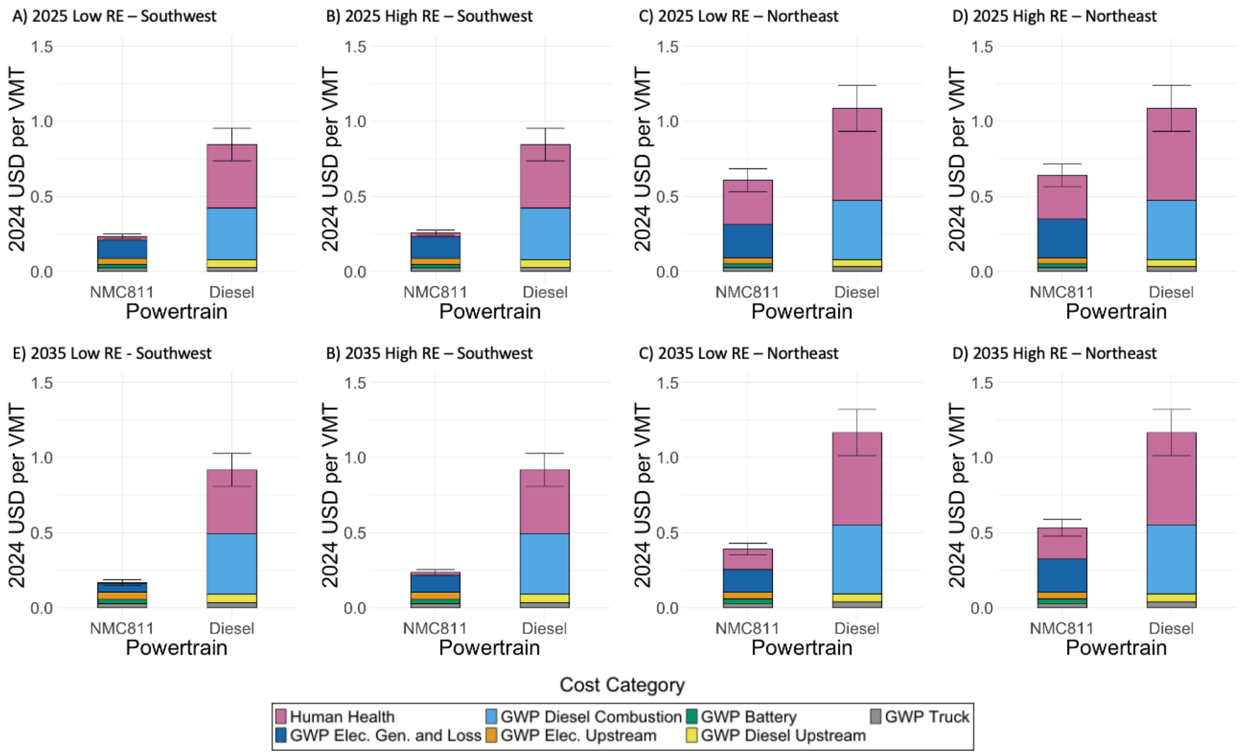

Fig S40. External costs of NMC811 BE-HDVs and diesel HDVs performing long-haul freight within different highway corridors with a 7% discount rate. (**A**) Visualized social costs on the southwest corridor in 2025 under a low renewable energy cost scenario. (**B**) Visualized social costs on the southwest corridor in 2025 under a high renewable energy cost scenario. (**C**) Visualized social costs on the northeast corridor in 2024 under a low renewable energy cost scenario. (**D**) Visualized social costs on the northeast corridor in 2024 under a high renewable energy cost scenario. (**E**) Visualized social costs on the southwest corridor in 2035 under a low renewable energy cost scenario. (**F**) Visualized social costs on the southwest corridor in 2035 under a high renewable energy cost scenario. (**G**) Visualized social costs on the northeast corridor in 2035 under a low renewable energy cost scenario. (**H**) Visualized social costs on the northeast corridor in 2035 under a high renewable energy cost scenario.

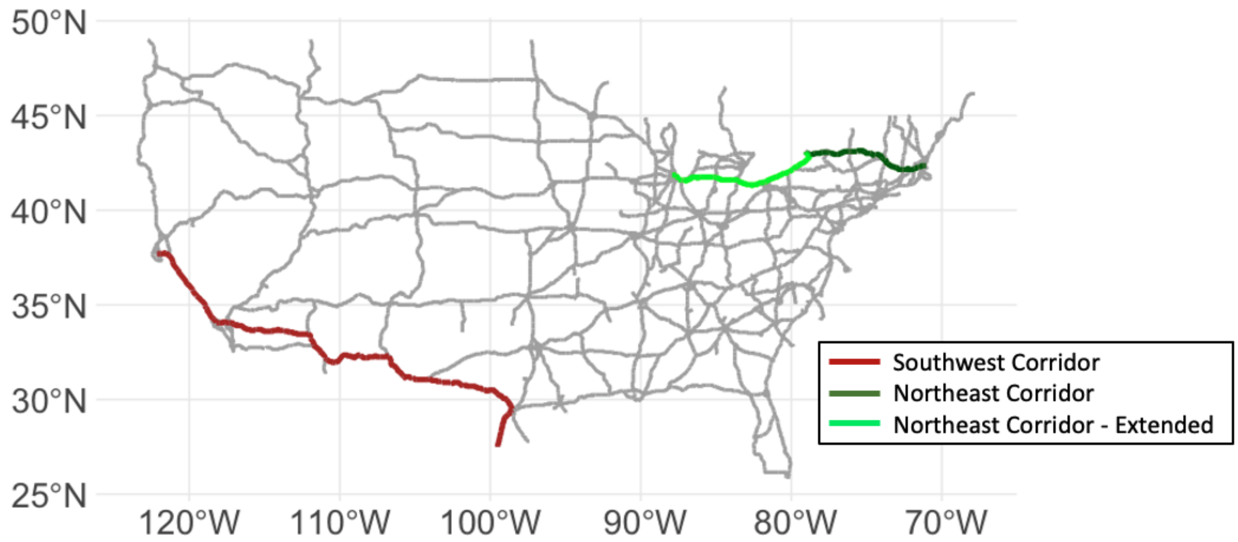

Fig S41. Southwest and northeast trucking corridors employed in the corridor sensitivity analysis. The southwest corridor is selected due to recent press on Tesla's plans to electrify said corridor for trucking.[26] The northeast corridor is selected as an extension of the Boston, MA to Buffalo, NY trucking corridor examined in Katsh et al.[27] This corridor was extended to Chicago to have a comparable length to the southwest corridor. When modeling trucking behavior along these corridors, major trucking destinations within 100 miles of the corridor are still permitted. Otherwise, the truck is unable to leave the corridor during trips. Geographic data on United States truck corridors is made available by the United States Bureau of Transportation Statistics.[28]

Table S4. Mass Breakdown of Truck Components[6,14,29,30]

Component	Diesel Truck Percentage	Li-ion Truck Percentage
**Truck Body**		
Body & Glass	5.8%	6.6%
Interior	6.3%	7.1%
Exterior	1.4%	1.6%
**Chassis**		
Steer Axle	3.1%	3.5%
Drive Axle	3.9%	4.4%
Shafts	4.6%	5.2%
Suspensions	4.5%	5.0%
Wheels and Tires	4.1%	4.7%
Cradle	8.9%	10.1%
**Powertrain**		
Engine	10.6%	0.0%
Engine Fuel Storage and Exhaust	1.7%	0.0%
**Electric Drive Components**		
Traction Motor	0.0%	2.9%
Electronic Controller	0.0%	0.3%
**Transmission**		
Clutch	0.2%	0.0%
Gearbox	3.1%	1.4%
Final Drive and Coupling	0.4%	0.4%
**Trailer**		
Trailer Body	24.1%	27.3%
Trailer Chassis	18.8%	21.3%
Trailer Auxiliary	2.6%	2.9%

Table S5. Distribution and Assumptions for Truck Component LCAs[30]

**Component/Parameter**	**Distribution/Characteristics**
Truck Body – Body and glass	Material Breakdown by Mass:<br>Glass fiber = 54%<br>Aluminum = 23%<br>Steel = 19%<br>LDPE = 25%
Truck Body - Interior	Material Breakdown by Mass:<br>Steel = 49%<br>LDPE = 25%<br>Fabric = 7%<br>Latex = 5%<br>Leather = 4%<br>Rubber = 3%<br>Aluminum = 6%
Truck Body - Exterior	Material Breakdown by Mass:<br>LDPE = 43%<br>Steel = 24%<br>Glass fiber = 10%<br>Aluminum = 10%<br>Rubber = 8%<br>Copper = 4%
Truck Chassis – Steer Axle	Material Breakdown by Mass:<br>Aluminum = 100%
Truck Chassis – Drive Axle	Material Breakdown by Mass:<br>Steel = 82%<br>Cast iron = 17%
Truck Chassis – Shafts	Material Breakdown by Mass:<br>Steel = 96%<br>Cast iron = 4%
Truck Chassis – Suspension	Material Breakdown by Mass:<br>Steel = 92%<br>Cast iron = 2%<br>Rubber = 6%
Truck Chassis – Wheels and tires	Material Breakdown by Mass:<br>Aluminum = 50%<br>Rubber = 33%<br>Steel = 17%

Truck Chassis – Cradle	Material Breakdown by Mass:<br>Steel = 98%<br>Rubber = 2%
Diesel Truck Powertrain - Engine	Material Breakdown by Mass:<br>Steel = 46%<br>Cast iron = 37%<br>Aluminum = 11%<br>LDPE = 3%<br>Copper = 3%
Diesel Truck Powertrain – Fuel Storage and Exhaust	Material Breakdown by Mass:<br>Aluminum = 35%<br>Ceramic = 23%<br>LDPE = 19%<br>Stainless steel = 13%<br>Steel = 10%
Li-ion Truck Electric Drive Components – Tractive Motor	Material Breakdown by Mass:<br>Steel = 36%<br>Aluminum = 36%<br>Copper = 28%
Li-ion Truck Electric Drive Components – Electronic Controller	Material Breakdown by Mass:<br>Steel = 5%<br>Aluminum = 47%<br>Copper = 8%<br>Rubber = 4%<br>LDPE = 24%
Diesel Truck Transmission - Clutch	Material Breakdown by Mass:<br>Steel = 86%<br>Cast iron = 7%<br>LDPE = 5%<br>Rubber = 1%
Diesel Truck Transmission - Gearbox	Material Breakdown by Mass:<br>Steel = 86%<br>Cast iron = 7%<br>LDPE = 5%<br>Rubber = 1%
Diesel Truck Transmission – Final Drive and Coupling	Material Breakdown by Mass:<br>Steel = 86%<br>Cast Iron = 7%<br>LDPE = 5%<br>Rubber = 1%

Li-ion Truck Transmission – Gearbox	Material Breakdown by Mass:<br>Steel = 86%<br>Cast iron = 7%<br>LDPE = 5%<br>Rubber = 1%
Li-ion Truck Transmission – Final Drive and Coupling	Material Breakdown by Mass:<br>Steel = 86%<br>Cast iron = 7%<br>LDPE = 5%<br>Rubber = 1%
Truck Trailer - Body	Material Breakdown by Mass:<br>Aluminum = 51%<br>Wood = 38%<br>Steel = 11%
Truck Trailer - Chassis	Material Breakdown by Mass:<br>Steel = 58%<br>Rubber = 18%<br>Cast iron = 14%<br>Aluminum = 9%
Truck Trailer - Auxiliary	Material Breakdown by Mass:<br>Steel = 69%<br>Glass fiber = 17%<br>Aluminum = 6%<br>Rubber = 5%<br>Copper = 3%
Truck Assembly Energy Demand (MJ)	Uniform distribution; min = 6255; max = 10425
Truck Assembly Energy Demand - Thermal	Thermal energy percent of total energy: triangle distribution; min = 0%, max = 80%, mode = 40%
Truck Assembly Energy Demand - Electricity	Electrical energy percent of total energy: 100% - thermal energy percent
Truck Assembly Thermal Source – Natural Gas	Thermal energy provided by natural gas: uniform distribution; min = 0%, max = 100%
Truck Assembly Thermal Source – Diesel	Thermal energy provided by natural gas: 100% - natural gas energy percent
Diesel Engine Assembly Energy Demand (MJ)	Uniform distribution; min = 682; max = 1137

Diesel Engine Assembly Energy Demand - Thermal	Thermal energy percent of total energy: triangle distribution; min = 0%, max = 80%, mode = 40%
Diesel Engine Assembly Energy Demand - Electricity	Electrical energy percent of total energy: 100% - thermal energy percent
Diesel Engine Assembly Thermal Source – Natural Gas	Thermal energy provided by natural gas: uniform distribution; min = 0%, max = 100%
Diesel Engine Assembly Thermal Source – Diesel	Thermal energy provided by natural gas: 100% - natural gas energy percent
Electric Motor Assembly Energy Demand (MJ)	Uniform distribution; min = 363; max = 604
Electric Motor Assembly Energy Demand - Thermal	Thermal energy percent of total energy: triangle distribution; min = 0%, max = 80%, mode = 40%
Electric Motor Assembly Energy Demand - Electricity	Electrical energy percent of total energy: 100% - thermal energy percent
Electric Motor Assembly Thermal Source – Natural Gas	Thermal energy provided by natural gas: uniform distribution; min = 0%, max = 100%
Electric Motor Assembly Thermal Source – Diesel	Thermal energy provided by natural gas: 100% - natural gas energy percent

Table S6. Uncertainty of Truck Model Parameters

Parameter	Uncertainty
R_{Br} [%]	+/- 1%, triangle distribution
h_{BW} [%]	+/- 1%, triangle distribution
h_{Br} [%]	+/- 1%, triangle distribution
h_{GB} [%]	+/- 1%, triangle distribution
h_E [%]	+/- 1%, triangle distribution
h_{TW} [%]	+/- 1%, triangle distribution
C_D	+/- 5%, triangle distribution
A [m^2]	+/- 5%, triangle distribution
C_{rr}	+/- 5%, triangle distribution
m_V [kg]	+/- 5%, triangle distribution
P_{AC} [kW]	+/- 5%, triangle distribution

Table S7. Li-ion Pack Specific Energy and Uncertainty by Cathode Chemistry.[31]

Cathode Chemistry	Specific Energy [Wh/kg]	Uncertainty
LFP	165	Triangle distribution: Min = 150, Max = 180
NMC*	255	Triangle distribution: Min = 250, Max = 260
NCA	177	Triangle distribution: Min = 172, Max = 188

* Assumed NMC811

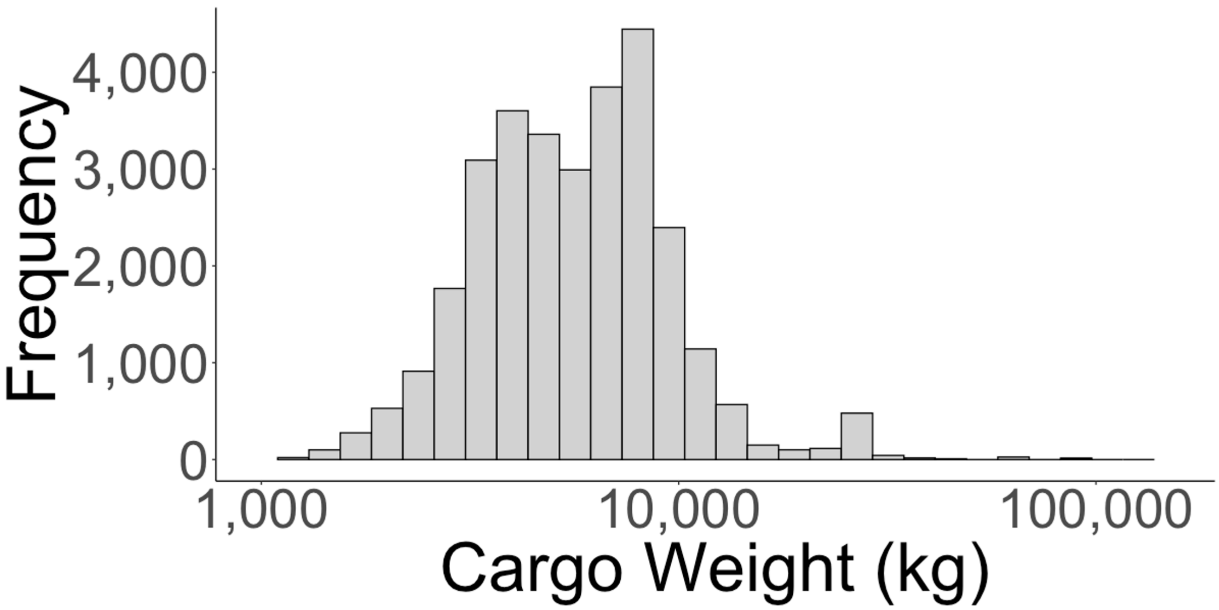

Fig. S42. Distribution of cargo weight for class 8 HDVs.[32]

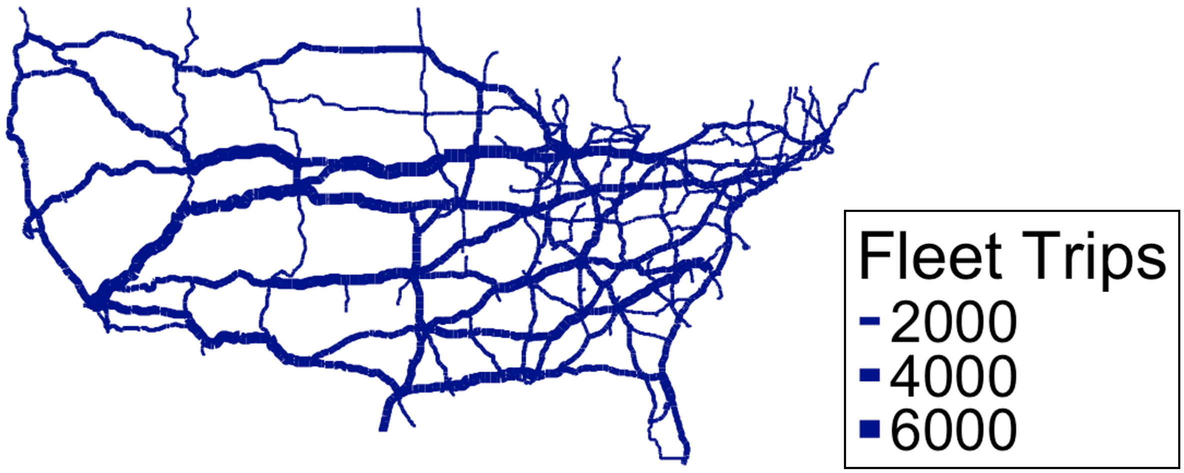

Fig. S43. Simulated HDV national flow.[32] Geographic data on United States truck corridors is made available by the United States Bureau of Transportation Statistics.[28]

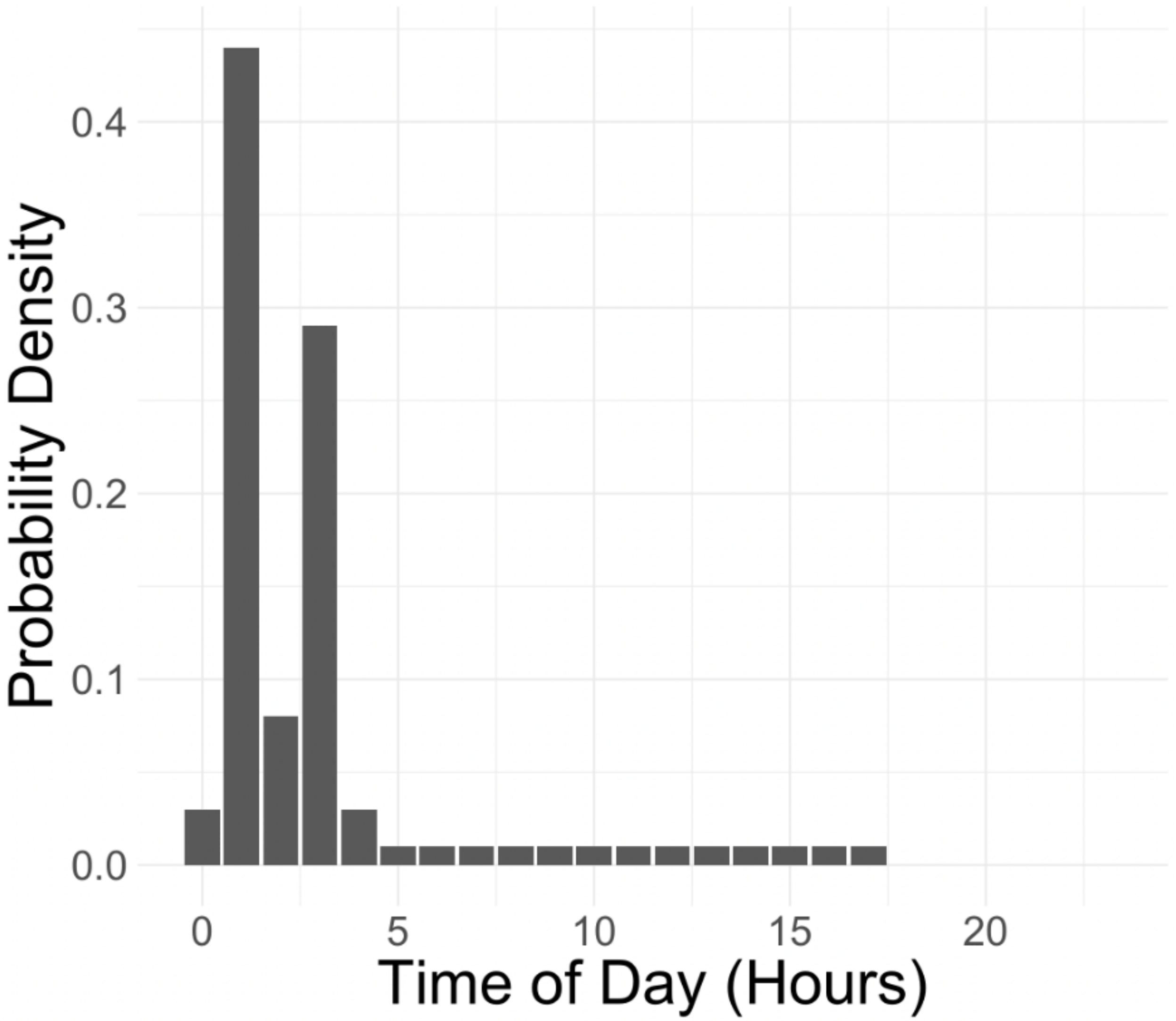

Fig S44. Discrete Probability Density of Initial Dispatch Times.[33]

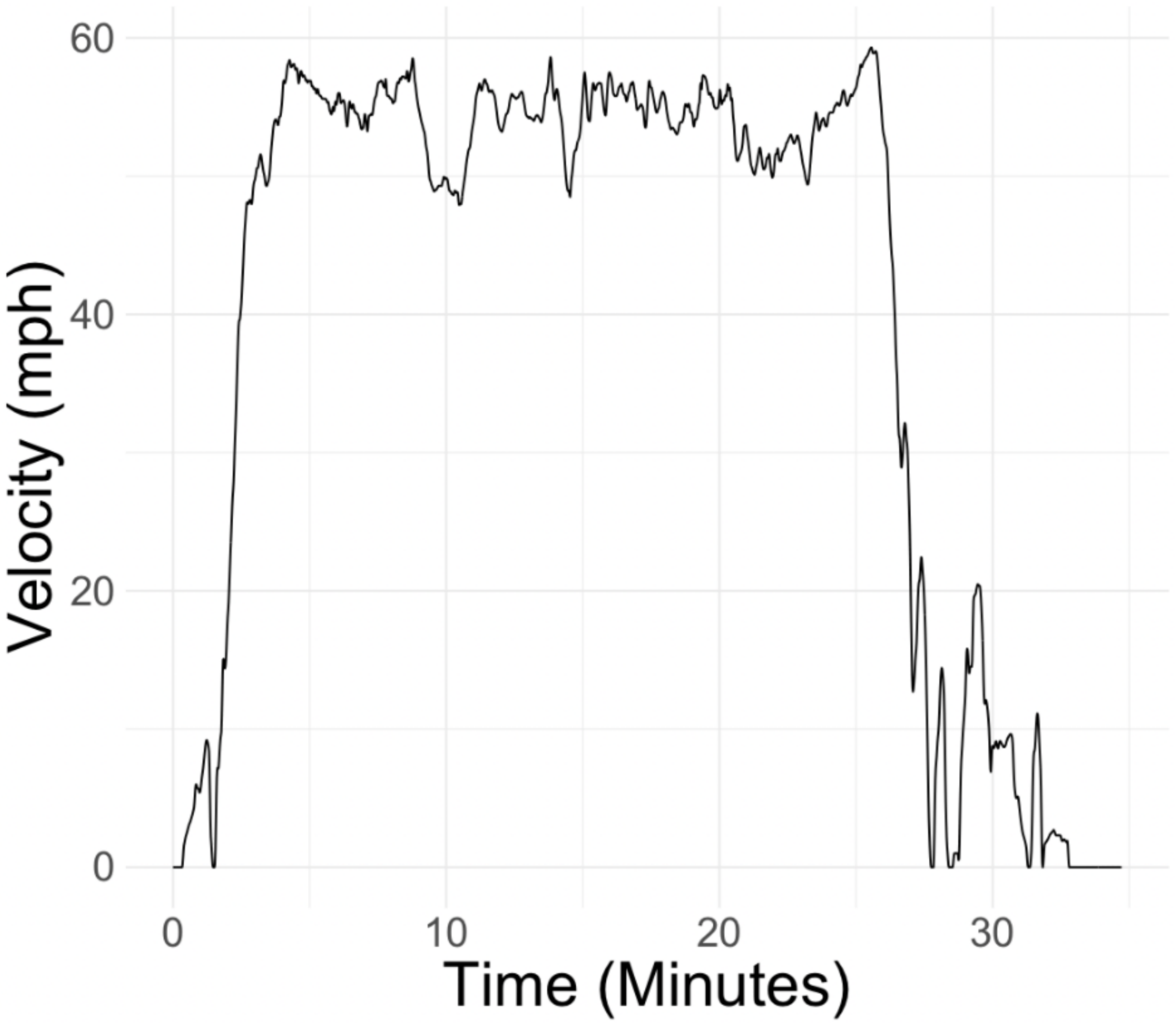

Fig S45. Class 8 Truck Drive Cycle - CARB Heavy Heavy-Duty Diesel Truck (CHHDDT) Cruise Segment.[34]

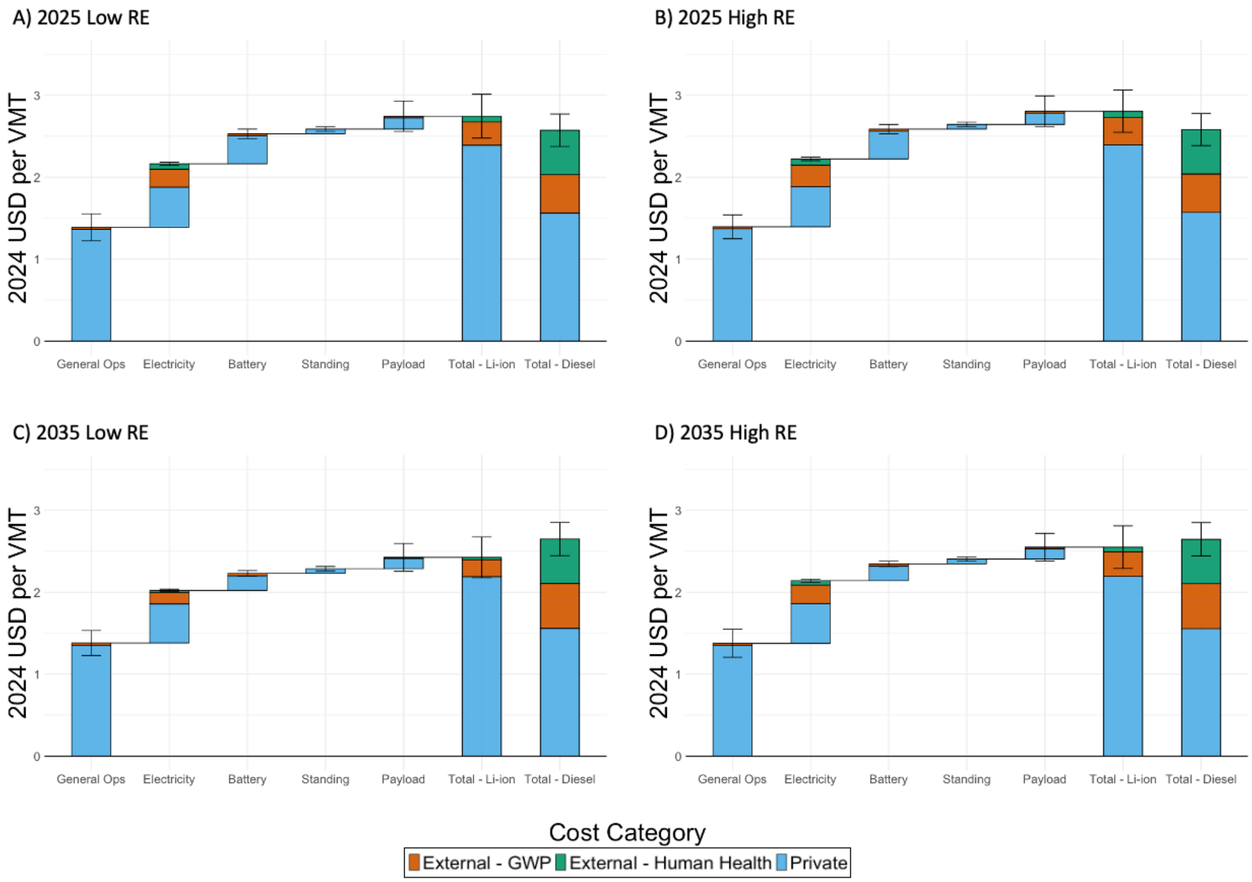

Fig. S46. Social costs with a simplified drive cycle (constant 65 mph).

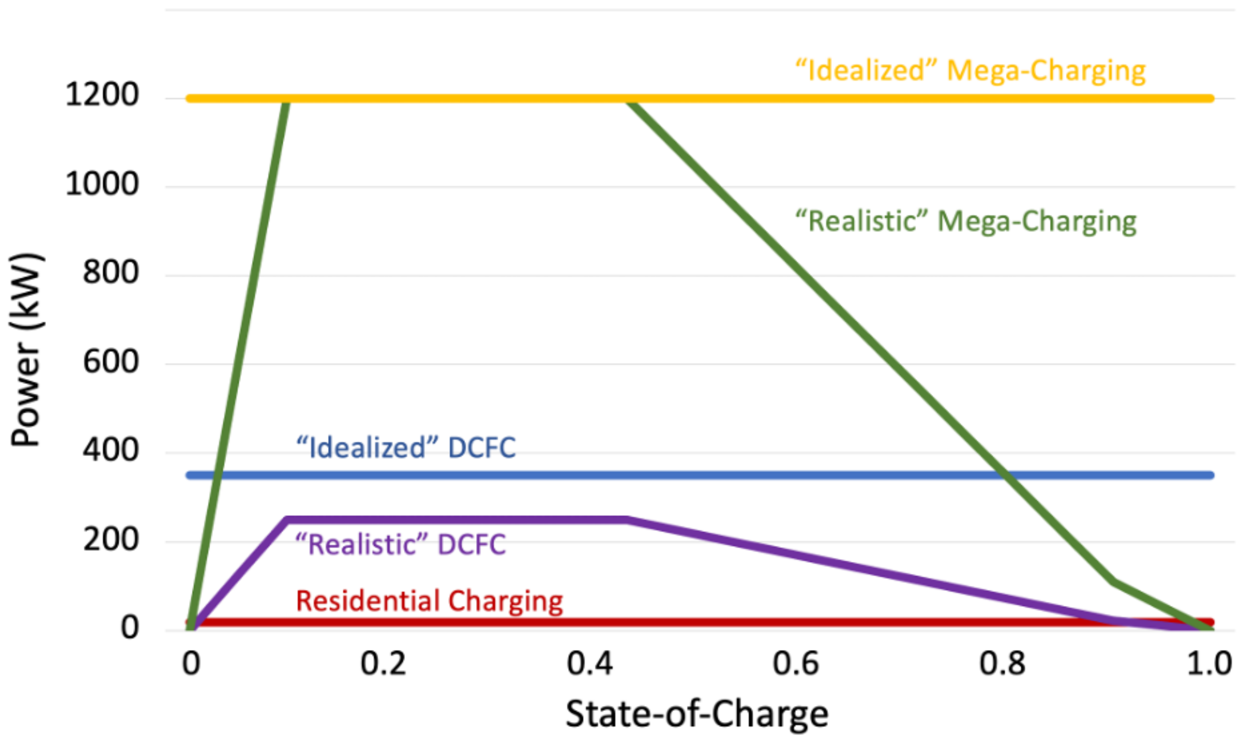

Fig S47. Modeled Charging Power vs State-of-Charge by Charging Infrastructure.[35–37]

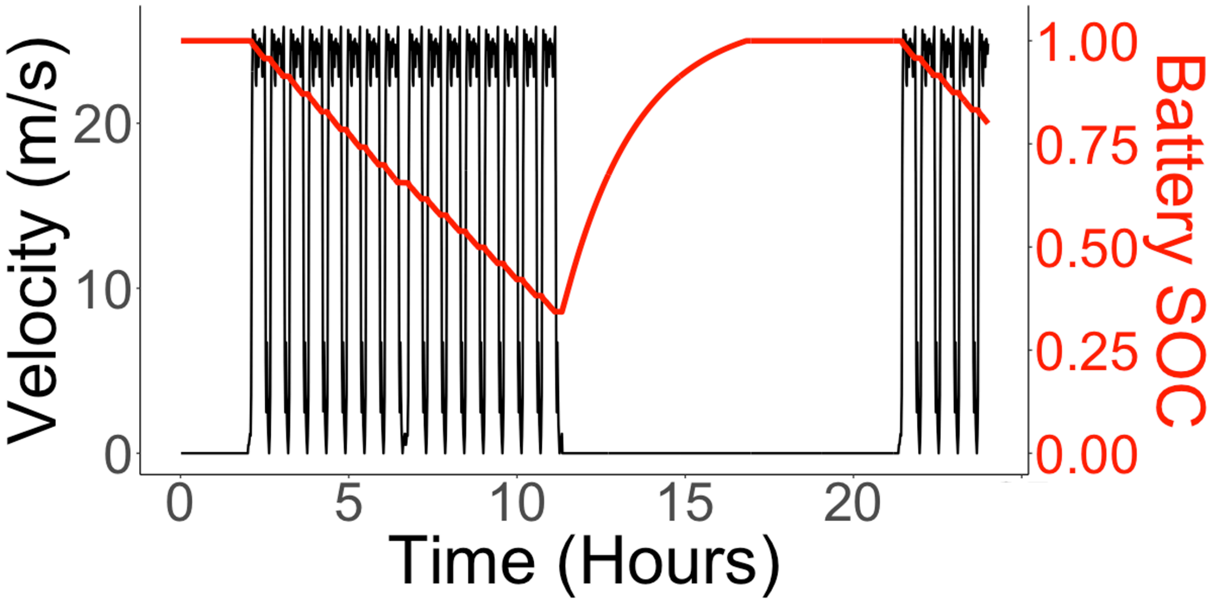

Fig. S48. One day example simulation of HDV velocity and battery state-of-charge (SOC).

Table S8. Tailpipe Emission Factors of Long-haul Diesel Trucks by Scenario[6]

**Scenario:**	Diesel Truck 2025	Diesel Truck 2035
**Vehicle Technology:**	MY 2010-2018	Advanced Design
**Pollution Control Technology:**	DPF* + SCR**	DPF + SCR
CO2 [g/kg-fuel]	3194	3194
CH4 [g/kg-fuel]	0.67	0.67
N2O [g/kg-fuel]	0.004	0.004
PM2.5 [g/kg-fuel]	0.3	0.3
SO2 [g/kg-fuel]	0.022	0.022
NOX [g/kg-fuel]	5.2	5.2
NH3 [g/kg-fuel]	0.18	0.18
Mile per Gallon	5.4	6.7

*DPF: Diesel particulate filter

**SCR: Selective catalytic reduction

Table S9. Class 8 Truck General Ops Cost Calculation and Uncertainty

Component/Parameter	Distribution/Characteristics	Source
Vehicle Depreciation	Lifetime Cost = MSRP * (1 – exp(A * lifetime + M * annual_VMT * lifetime / 1000) * (1 – r)^lifetime<br><br>MSRP: Uniform Distribution; min = 100,000; max = 140,000<br>A = log(0.9071)<br>M = log(0.9990)<br>r = discount rate	[13]
Insurance	Lifetime Cost =<br><br>ins_per_mile: Triangle distribution; min = 0.06292; max = 0.09438; mode = 0.07865<br>r = discount rate	[14]
Taxes Fixed	Lifetime Cost = (MSRP + Battery Cost) * 0.12	[38]
Taxes Annual	Lifetime Cost =<br><br>r = discount rate	[39]
Fees Annual	Lifetime Cost =<br><br>fees_per_kg: Uniform distribution; min = 0.04545; max = 0.06363<br>fees_fixed = 5952<br>r = discount rate	[13,14]
Maintenance and Repairs	Lifetime Cost =<br><br>M = 0.03<br>b = 0.09<br>Batt_Adj: if diesel, Batt_Adj = 1<br>If Li-ion, Triangle distribution; min = 0.48; max = 0.72; mode = 0.6<br>r = discount rate	[13,14]
Labor Driving	Lifetime Cost =<br><br>lab_per_mile: Triangle distribution; min = 0.7837; max = 1.176; mode = 0.9796<br>r = discount rate	[13,14]

annual_VMT: annual vehicle miles traveled

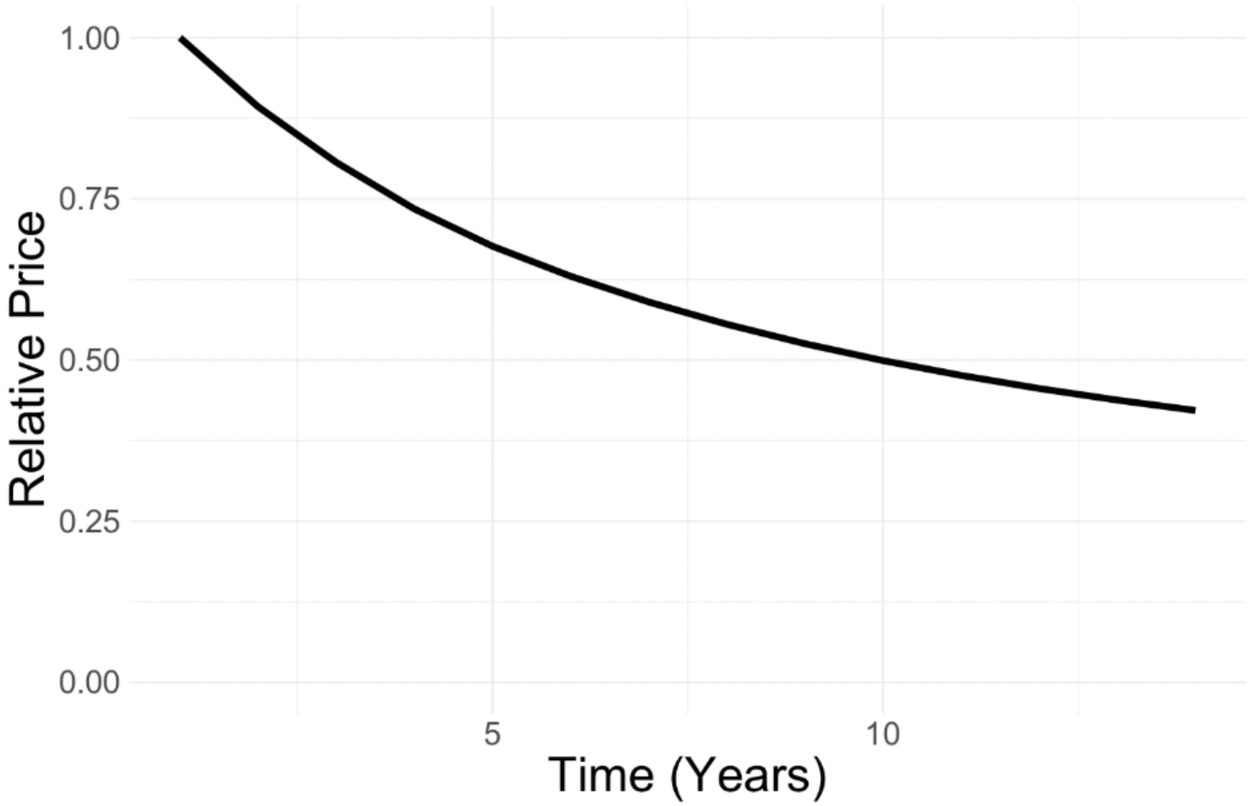

Fig S49. **Forecasted battery prices with 17.3% learning rate**[40,41] Demand growth in Table S11 used to forecast relative battery prices.

Tables S10. Forecasted Global Li-ion Battery Demand Scenario[40]

Year	Demand (GWh)
2022	1611
2023	2456
2024	3582
2025	5079
2026	6892
2027	8987
2028	11463
2029	14348
2030	17657
2031	21358
2032	25452
2033	29931
2034	34774
2035	39955

Table S11. 2022 Li-ion Battery Pack Triangular Distribution Prices ($/kWh) by Chemistry in USD_{2024}[31]

**Chemistry**	**Min**	**Max**	**Mode**
NMC811	156	234	195
NCA	129	193	161
LFP	114	172	143

Table S12. Average US electricity generation marginal emission factors, excluding solar and wind generators, by scenario.

**Scenario**	**NO_x (g/kWh)**	**SO_2 (g/kWh)**	**N_2O (g/kWh)**	**CH_4 (g/kWh)**	**$PM_{2.5}$ (g/kWh)**	**CO_2 (kg/kWh)**
LowRE 2025	0.4350	0.7553	0.0140	0.0998	0.1110	0.4786
LowRE 2035	0.2554	0.3337	0.0074	0.0534	0.0629	0.2632
HighRE 2025	0.5081	0.8281	0.0155	0.1101	0.1151	0.5824
HighRE 2035	0.4153	0.5933	0.0133	0.0955	0.1085	0.4383

Table S13. Average US marginal emissions per total electricity demand from battery charging by scenario.

**Scenario**	**NO_x (g/kWh)**	**SO_2 (g/kWh)**	**N_2O (g/kWh)**	**CH_4 (g/kWh)**	**$PM_{2.5}$ (g/kWh)**	**CO_2 (kg/kWh)**
LowRE 2025	0.4113	0.7142	0.0133	0.0944	0.1049	0.4526
LowRE 2035	0.1945	0.2541	0.0056	0.0407	0.0479	0.2004
HighRE 2025	0.4932	0.8038	0.0151	0.1079	0.1181	0.5654
HighRE 2035	0.3686	0.5264	0.0118	0.0848	0.0962	0.3889

References

1. Siler-Evans, K., Azevedo, I. L. & Morgan, M. G. Marginal emissions factors for the U.S. electricity system. *Environ. Sci. Technol.* **46**, 4742–4748 (2012).

2. Graff Zivin, J. S., Kotchen, M. J. & Mansur, E. T. Spatial and temporal heterogeneity of marginal emissions: Implications for electric cars and other electricity-shifting policies. *J. Econ. Behav. Organ.* **107**, 248–268 (2014).

3. Archsmith, J., Kendall, A. & Rapson, D. From cradle to junkyard: assessing the life cycle greenhouse gas benefits of electric vehicles. *Research in Transportation Economics* **52**, 72–90 (2015).

4. Hoehne, C. G. & Chester, M. V. Optimizing plug-in electric vehicle and vehicle-to-grid charge scheduling to minimize carbon emissions. *Energy* **115**, 646–657 (2016).

5. Jenn, A., Clark-Sutton, K., Gallaher, M. & Petrusa, J. Environmental impacts of extreme fast charging. *Environmental Research Letters* **15**, 094060 (2020).

6. Tong, F., Jenn, A., Wolfson, D., Scown, C. D. & Auffhammer, M. Health and Climate Impacts from Long-Haul Truck Electrification. *Environ. Sci. Technol.* **55**, 8514–8523 (2021).

7. McNeil, W. H., Tong, F., Harley, R. A., Auffhammer, M. & Scown, C. D. Corridor-Level Impacts of Battery-Electric Heavy-Duty Trucks and the Effects of Policy in the United States. *Environ. Sci. Technol.* **58**, 33–42 (2024).

8. McNeil, W. H. *et al.* Impact of truck electrification on air pollution disparities in the United States. *Nat. Sustain.* (2025) doi:10.1038/s41893-025-01515-x.

9. Gagnon, P. & Cole, W. Planning for the evolution of the electric grid with a long-run marginal emission rate. *iScience* **25**, 103915 (2022).

10. Ryan, N. A., Johnson, J. X. & Keoleian, G. A. Comparative assessment of models and methods to calculate grid electricity emissions. *Environ. Sci. Technol.* **50**, 8937–8953 (2016).

11. Holland, S. P., Kotchen, M. J., Mansur, E. T. & Yates, A. J. Why marginal CO2 emissions are not decreasing for US electricity: Estimates and implications for climate policy. *Proc Natl Acad Sci USA* **119**, (2022).

12. Bruchon, M., Chen, Z. L. & Michalek, J. Cleaning up while Changing Gears: The Role of Battery Design, Fossil Fuel Power Plants, and Vehicle Policy for Reducing Emissions in the Transition to Electric Vehicles. *Environ. Sci. Technol.* **58**, 3787–3799 (2024).

13. Hunter, C. *et al. Spatial and Temporal Analysis of the Total Cost of Ownership for Class 8 Tractors and Class 4 Parcel Delivery Trucks*. (2021).

14. Burnham, A. *et al. Comprehensive Total Cost of Ownership Quantification for Vehicles with Different Size Classes and Powertrains*. (2021).

15. Basma, H., Buysse, C., Zhou, Y. & Rodriguez, F. *Total Cost of Ownership of Alternative Powertrain Technologies for Class 8 Long-haul Trucks in the United States*. (2023).

16. Rout, C., Li, H., Dupont, V. & Wadud, Z. A comparative total cost of ownership analysis of heavy duty on-road and off-road vehicles powered by hydrogen, electricity, and diesel. *Heliyon* **8**, e12417 (2022).

17. Phadke, A., Khandekar, A., Abhyankar, N., Wooley, D. & Rajagopal, D. *Why Regional and Long-Haul Trucks are Primed for Electrification Now*. (2021).

18. Forkenbrock, D. J. Comparison of external costs of rail and truck freight transportation. *Transportation Research Part A: Policy and Practice* **35**, 321–337 (2001).

19. Syré, A. M. & Göhlich, D. Decarbonization of Long-Haul Heavy-Duty Truck Transport: Technologies, Life Cycle Emissions, and Costs. *WEVJ* **16**, 76 (2025).

20. Burke, A. F., Zhao, J., Miller, M. R., Sinha, A. & Fulton, L. M. Projections of the costs of medium- and heavy-duty battery-electric and fuel cell vehicles (2020-2040) and related economic issues. *Energy for Sustainable Development* **77**, 101343 (2023).

21. Zhao, P. *et al.* Challenges and opportunities in truck electrification revealed by big operational data. *Nat. Energy* **9**, 1427–1437 (2024).

22. Cohon, J. L. *et al. Hidden costs of energy: unpriced consequences of energy production and use*. (National Academies Press, 2010). doi:10.17226/12794.

23. Kemperdick, T. & Letmathe, P. External costs of battery-electric and fuel cell electric vehicles for heavy-duty applications. *Transportation Research Part D: Transport and Environment* **131**, 104198 (2024).

24. Austin, D. *Pricing Freight Transport to Account for External Costs*. (2015).

25. US EPA. *Supplementary Material for the Regulatory Impact Analysis for the Final Rulemaking, "Standards of Performance for New, Reconstructed, and Modified Sources and Emissions Guidelines for Existing Sources: Oil and Natural Gas Sector Climate Review" - EPA Report on the Social Cost of Greenhouse Gases: Estimates Incorporating Recent Scientific Advances*. (2023).

26. O'Kane, S. & Hull, D. Tesla Wants to Build a Semi Truck-Charging Route From Texas to California. *Bloomberg* https://www.bloomberg.com/news/articles/2023-08-01/tesla-semi-truck-charging-route-pitched-at-100-million (2023).

27. Katsh, G. *et al. Electric Highways: Accelerating and Optimizing Fast-Charging Deployment for Carbon-Free Transportation*. (2022).

28. United States Bureau of Transportation Statistics. National Transportation Atlas Database. *Bureau of Transportation Statistics - Open Data Catalog* https://data-usdot.opendata.arcgis.com/ (2024).

29. Hunter, C. A. *et al.* Techno-economic analysis of long-duration energy storage and flexible power generation technologies to support high-variable renewable energy grids. *Joule* (2021) doi:10.1016/j.joule.2021.06.018.

30. Wang, M. *GREET (Greenhouse Gases, Regulated Emissions, and Energy Use in Transportation) Model*. (Argonne National Laboratory, 2023).

31. BloombergNEF. *2022 Lithium-Ion Battery Price Survey*. (2022).

32. US Department of Transportation, Bureau of Transportation Statistics, US Department of Commerce & US Census Bureau. 2017 Commodity Flow Survey Datasets. https://www.census.gov/data/datasets/2017/econ/cfs/historical-datasets.html (2020).

33. Boriboonsomsin, K. *et al. Collection of Activity Data from On-Road Heavy-Duty Diesel Vehicles*. (2017).

34. National Renewable Energy Laboratory. NREL DriveCAT - Chassis Dynamometer Drive Cycles. https://www.nrel.gov/transportation/drive-cycle-tool/ (2023).

35. Andrenacci, N., Karagulian, F. & Genovese, A. Modelling charge profiles of electric vehicles based on charges data. *Open Res. Europe* **1**, 156 (2021).

36. Hackmann, M. *P3 Charging Index Report 07/22 – Comparison of the Fast Charging Capability of Various Electric Vehicles*. (2022).

37. Moura, S. Interview with Prof. Scott Moura. (2023).

38. US EPA. Learn About Federal Excise Tax Exemption. https://www.epa.gov/verified-diesel-tech/learn-about-federal-excise-tax-exemption (2023).

39. US Department of Transportation. Heavy Vehicle Use Tax. *Policy and Governmental Affairs Office of Highway Policy Information* https://www.fhwa.dot.gov/policyinformation/hvut/mod1/whatishvut.cfm (2020).

40. BloombergNEF. *Lithium-Ion Batteries: State of the Industry 2023*. (2023).

41. Ziegler, M. & Trancik, J. *Re-examining Rates of Lithium-ion Battery Technology Improvement and Cost Declines*. (2020).
