# Supplementary material for: Electrifying long-haul freight trucks reduces societal costs in the United States
Source: Nat Commun. 2025 Dec 12;17:468. doi: 10.1038/s41467-025-67161-1 (PMC12800144; doi:10.1038/s41467-025-67161-1)
Supplement: Supplementary file 2 — Reporting Summary [file 41467_2025_67161_MOESM2_ESM.pdf]

nature portfolio

Corresponding author(s): Corinne D. Scown, Maximilian Auffhammer

Last updated by author(s): Oct 28, 2025

Reporting Summary

Nature Portfolio wishes to improve the reproducibility of the work that we publish. This form provides structure for consistency and transparency in reporting. For further information on Nature Portfolio policies, see our Editorial Policies and the Editorial Policy Checklist.

Statistics

For all statistical analyses, confirm that the following items are present in the figure legend, table legend, main text, or Methods section.

n/a	Confirmed	
☐	☒	The exact sample size (n) for each experimental group/condition, given as a discrete number and unit of measurement
☒	☐	A statement on whether measurements were taken from distinct samples or whether the same sample was measured repeatedly
☒	☐	The statistical test(s) used AND whether they are one- or two-sided<br>*Only common tests should be described solely by name; describe more complex techniques in the Methods section.*
☒	☐	A description of all covariates tested
☒	☐	A description of any assumptions or corrections, such as tests of normality and adjustment for multiple comparisons
☐	☒	A full description of the statistical parameters including central tendency (e.g. means) or other basic estimates (e.g. regression coefficient) AND variation (e.g. standard deviation) or associated estimates of uncertainty (e.g. confidence intervals)
☒	☐	For null hypothesis testing, the test statistic (e.g. F, t, r) with confidence intervals, effect sizes, degrees of freedom and P value noted<br>*Give P values as exact values whenever suitable.*
☒	☐	For Bayesian analysis, information on the choice of priors and Markov chain Monte Carlo settings
☒	☐	For hierarchical and complex designs, identification of the appropriate level for tests and full reporting of outcomes
☒	☐	Estimates of effect sizes (e.g. Cohen's d, Pearson's r), indicating how they were calculated

Our web collection on statistics for biologists contains articles on many of the points above.

Software and code

Policy information about availability of computer code

Data collection	No custom code or software was used for data collection in this study. All data it collected from publicly available sources made available in the Data Availability statement.
Data analysis	RStudio version 2025.05.0+496, R version 4.5.0, Jupyter Notebook version 7.3.2, and Python version 3.11.5 are used to perform data analysis. Code is made available in the Code Availability statement.

For manuscripts utilizing custom algorithms or software that are central to the research but not yet described in published literature, software must be made available to editors and reviewers. We strongly encourage code deposition in a community repository (e.g. GitHub). See the Nature Portfolio guidelines for submitting code & software for further information.

Data

Policy information about availability of data

All manuscripts must include a data availability statement. This statement should provide the following information, where applicable:

- Accession codes, unique identifiers, or web links for publicly available datasets
- A description of any restrictions on data availability
- For clinical datasets or third party data, please ensure that the statement adheres to our policy

The processed Figure 1-4 data is available at https://doi.org/10.6084/m9.figshare.28673372. The freight truck counts data are available from the 2017 Commodity Flow Survey Datasets at https://www.census.gov/data/datasets/2017/econ/cfs/historical-datasets.html (ref. 62). The electricity grid emissions calculations data are available from the National Renewable Energy Laboratory's Cambium tool at https://www.nrel.gov/analysis/cambrium.html (ref. 69 & 72). The Source-Receptor

Matrices used for health impacts estimation data are available at https://inmap.run/. The representative drive cycle data are available from the National Renewable Energy Laboratory's DriveCAT: Drive Cycle Analysis Tool at https://www.nrel.gov/transportation/drive-cycle-tool (ref. 65).

Research involving human participants, their data, or biological material

Policy information about studies with human participants or human data. See also policy information about sex, gender (identity/presentation), and sexual orientation and race, ethnicity and racism.

Reporting on sex and gender	NA
Reporting on race, ethnicity, or other socially relevant groupings	NA
Population characteristics	NA
Recruitment	NA
Ethics oversight	NA

Note that full information on the approval of the study protocol must also be provided in the manuscript.

Field-specific reporting

Please select the one below that is the best fit for your research. If you are not sure, read the appropriate sections before making your selection.

☐ Life sciences ☐ Behavioural & social sciences ☒ Ecological, evolutionary & environmental sciences

For a reference copy of the document with all sections, see nature.com/documents/nr-reporting-summary-flat.pdf

Ecological, evolutionary & environmental sciences study design

All studies must disclose on these points even when the disclosure is negative.

Study description	This work models the private and monetized climate and health damages of electrifying long-haul heavy-duty diesel trucks with Li-ion batteries. This is achieved by simulating representative trucking behavior for battery electric and diesel trucks across the United states, determining their corresponding electricity generation response, and estimating the damages associated with their emissions from either the relevant generators or the their tailpipe.
Research sample	The research sample consists of 100 year long simulated vehicle behaviors via Marchov Chain Monte Carlo analysis for battery electric and diesel long-haul heavy duty trucks each (200 total simulations). Freight truck counts data are available from the 2017 Commodity Flow Survey Datasets at https://www.census.gov/data/datasets/2017/econ/cfs/historical-datasets.html which surveyed truck operation in 2017 and is used to inform truck origin-destination pairs and trip characteristics. Electricity grid emissions calculations data are available from the National Renewable Energy Laboratory's Cambium tool at https://www.nrel.gov/analysis/cambrium.html which observes emissions from individual electricity generation units and are used to inform electricity generation response. Source-Receptor Matrices used for health impacts estimation data are available at https://inmap.run/ which consists of census data and background concentrations and are used to inform human health damages from induced emissions. Representative drive cycle data are available from the National Renewable Energy Laboratory's DriveCAT: Drive Cycle Analysis Tool at https://www.nrel.gov/transportation/drive-cycle-tool which are taken from relevant governing bodies (e.g. CARB) and are used to inform truck behavior at a minute resolution.
Sampling strategy	OD pairs and trip characteristic were sampled from the 2017 Commodity Flow Survey (CFS) by generating PDFs of all possible destinations greater than 250 miles away (long-haul) and trip characteristics for a set origin using the "weight" variable provided by the CFS which represents the frequency of a given OD pair and trip characteristic occurring. The destination and trip characteristics were then randomly selected according to the weighting of the PDF for a given origin. The destination selected was then used as the origin for the next trip until a year of truck behavior had been simulated. Initial origins were randomly selected within the network of OD pairs. The behavior of 100 year long simulations was generated for both diesel and battery electric vehicles, representing a final number of individual trips at the order of magnitude of 10,000, appropriately weighted across the United States.
Data collection	Data was collected from publically available datasets, made available to the reader in the Data Availability statement
Timing and spatial scale	Commodity Flow Survey data is representative of truck behavior for the year 2017. This is the most up-to-date data at the national scale. Truck behavior was simulated at a single minute time resolution and a single kilometer spatial resolution. Electricity generation response is simulated per balancing area and T-region for the year 2025 and forecast into the year 2035. The ISRM used to calculate human health damages employs data from 2011 but is forecast using precedent established in literature to estimate damages for 2025 and 2035.
Data exclusions	No data exclusions are present in this analysis.
Reproducibility	All data is publicly available and methodology has been thoroughly described and documented, with code made available in the Code Availability statement.

Randomization	Origin destination pairs and trip characteristics are randomly selected via PDF generated from the recorded frequency OD pairs and trip characteristics available in the 2017 Freight Commodity Survey.
Blinding	Blinding is not relevant to this study given that truck behavior was simulated and weighted entirely by the historical frequency of occurrences.

Did the study involve field work? ☐ Yes ☒ No

Reporting for specific materials, systems and methods

We require information from authors about some types of materials, experimental systems and methods used in many studies. Here, indicate whether each material, system or method listed is relevant to your study. If you are not sure if a list item applies to your research, read the appropriate section before selecting a response.

Materials & experimental systems

n/a	Involved in the study
☒	☐ Antibodies
☒	☐ Eukaryotic cell lines
☒	☐ Palaeontology and archaeology
☒	☐ Animals and other organisms
☒	☐ Clinical data
☒	☐ Dual use research of concern
☒	☐ Plants

Methods

n/a	Involved in the study
☒	☐ ChIP-seq
☒	☐ Flow cytometry
☒	☐ MRI-based neuroimaging

Plants

Seed stocks	NA
Novel plant genotypes	NA
Authentication	NA
